# Novel insights into salinity-induced lipogenesis and carotenogenesis in the oleaginous astaxanthin-producing alga *Chromochloris zofingiensis*: a multi-omics study

**DOI:** 10.1186/s13068-020-01714-y

**Published:** 2020-04-16

**Authors:** Xuemei Mao, Yu Zhang, Xiaofei Wang, Jin Liu

**Affiliations:** Laboratory for Algae Biotechnology & Innovation, College of Engineering, Peking University, Beijing, 100871 China

**Keywords:** Astaxanthin, Alga, Carotenogenesis, Lipid metabolism, Salt stress, Triacylglycerol

## Abstract

**Background:**

*Chromochloris zofingiensis*, a freshwater alga capable of synthesizing both triacylglycerol (TAG) and astaxanthin, has been receiving increasing attention as a leading candidate producer. While the mechanism of oleaginousness and/or carotenogenesis has been studied under such induction conditions as nitrogen deprivation, high light and glucose feeding, it remains to be elucidated in response to salt stress, a condition critical for reducing freshwater footprint during algal production processes.

**Results:**

Firstly, the effect of salt concentrations on growth, lipids and carotenoids was examined for *C. zofingiensis*, and 0.2 M NaCl demonstrated to be the optimal salt concentration for maximizing both TAG and astaxanthin production. Then, the time-resolved lipid and carotenoid profiles and comparative transcriptomes and metabolomes were generated in response to the optimized salt concentration for congruent analysis. A global response was triggered in *C. zofingiensis* allowing acclimation to salt stress, including photosynthesis impairment, ROS build-up, protein turnover, starch degradation, and TAG and astaxanthin accumulation. The lipid metabolism involved a set of stimulated biological pathways that contributed to carbon precursors, energy and reductant molecules, pushing and pulling power, and storage sink for TAG accumulation. On the other hand, salt stress suppressed lutein biosynthesis, stimulated astaxanthin biosynthesis (mainly via ketolation), yet had little effect on total carotenoid flux, leading to astaxanthin accumulation at the expense of lutein. Astaxanthin was predominantly esterified and accumulated in a well-coordinated manner with TAG, pointing to the presence of common regulators and potential communication for the two compounds. Furthermore, the comparison between salt stress and nitrogen deprivation conditions revealed distinctions in TAG and astaxanthin biosynthesis as well as critical genes with engineering potential.

**Conclusions:**

Our multi-omics data and integrated analysis shed light on the salt acclimation of *C. zofingiensis* and underlying mechanisms of TAG and astaxanthin biosynthesis, provide engineering implications into future trait improvements, and will benefit the development of this alga for production uses under saline environment, thus reducing the footprint of freshwater.

## Introduction

Alternative energy sources to petroleum-based fuels have long been pursued, of which biofuels from alga, the next-generation feedstock, have received increasing interest of both academia and industry [1–3]. Despite the progresses achieved during the past decades, challenges remain yet to be addressed for bringing down the production cost and realizing commercialization of algal biofuels [4–6]. Among the strategies proposed for addressing challenges, integrated production of lipids with value-added products from algae is believed to be promising to improve algal biofuel production economics [7]. These products include, but are not restricted to high-value proteins (e.g., phycobilins), ω-3 polyunsaturated fatty acids (e.g., eicosapentaenoic acid and docosahexaenoic acid), and carotenoids (e.g., β-carotene, fucoxanthin and astaxanthin), depending on the source of algal species/strains [7]. It is worth noting that, from a biorefinery point of view, concurrent synthesis of value-added products and lipids by algae is a prerequisite for implementation of the integrated production concept. Triacylglycerol (TAG), the ideal lipid for making biodiesel, generally accumulates in algae under stress conditions [1]. Among the above-mentioned value-added products, astaxanthin represents a high-value carotenoid with broad industrial applications and tends to accumulate in certain algae upon these TAG-induction stresses [8–13], pointing to the feasibility of using algae for integrated production of the two compounds.

*Chromochloris zofingiensis*, also referred to as *Chlorella zofingiensis*, is a freshwater green alga capable of growing robustly under multiple trophic conditions, reaching up to 10 and 100 g L^−1^ for photoautotrophic and heterotrophic modes, respectively [9, 11, 12, 14]. *C. zofingiensis* synthesizes a high level of intracellular TAG (up to 50% dry weight) and has been cited as a potential feedstock for biodiesel [11, 12, 14, 15]. The alga is also able to synthesize high-value ketocarotenoids and is thought to be a candidate astaxanthin producer alternative to *Haematococcus pluvialis* [16]. The robustness in concurrent accumulation of TAG and astaxanthin [11, 14, 17] and availability of chromosome-level genome sequence [18] enable *C. zofingiensis* as an emerging model alga for both fundamental studies and industrial applications. While many studies deal with the engineering of culture conditions for TAG and astaxanthin production by *C. zofingiensis* [9, 11, 12, 14, 17, 19–22], the molecular mechanisms underlying their biosynthesis are less touched and remain to be fully explored in a system-level manner.

*Chromochloris zofingiensis* has the capacity to synthesize both TAG and astaxanthin in response to such cues as the deprivation of nutrients (e.g., nitrogen and sulfur), high light, and glucose induction [11, 14, 17, 23, 24]. The shortage of freshwater resources has led to studying the response of freshwater algae to salt and potential of utilizing seawater for production applications [25–29]. It has been reported that *C. zofingiensis* can tolerate moderate salt concentrations and accumulate astaxanthin as a response [9]. By contrast, the effect of salt stress on TAG synthesis by the alga remains to be evaluated. Recently, several transcriptomic studies have been performed for *C. zofingiensis* under the conditions of nitrogen deprivation, high light and glucose feeding [15, 18, 24, 30], contributing to the understanding of biosynthesis of TAG and/or astaxanthin. However, the data under salt stress conditions are still lacking for *C. zofingiensis* and the underlying mechanisms are yet to be disclosed. To fill the gap, here we optimized the salt concentrations for maximizing both TAG and astaxanthin production by *C. zofingiensis*, generated comparative transcriptomes and metabolomes, and determined the time-course profiles of lipids, carotenoids and other compounds. The congruent analysis of these large data sets shed light on the mechanisms of salt stress-associated oleaginousness and carotenogenesis in the emerging model alga *C. zofingiensis*, identifies potential limiting factors for TAG and astaxanthin accumulation, and provides useful implications into future genetic engineering of this alga for trait improvements.

## Results

### Optimization of salinity levels for lipid and astaxanthin production by *C. zofingiensis*

**Fig. 1:**
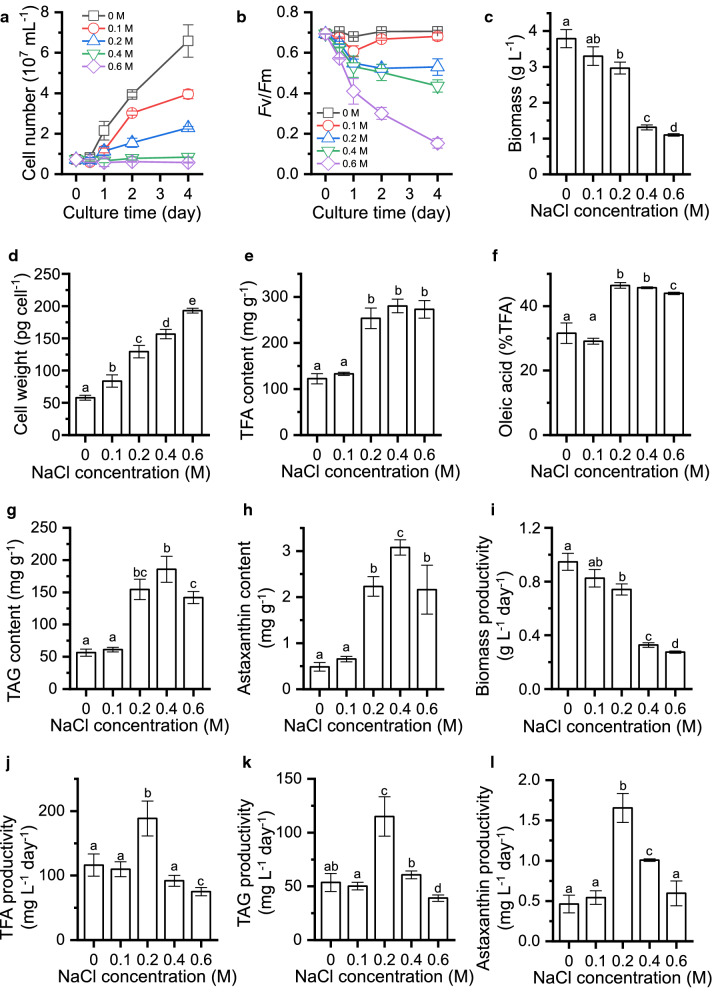
The physiological and biochemical changes affected by various NaCl concentrations. **a** Cell number. **b** *F*v/*F*m. **c** Biomass concentration. **d** Cell weight. **e** TFA content. **f** Oleic acid abundance. **g** TAG content. **h** Astaxanthin content. **i** Biomass productivity. **j** TFA productivity. **k** TAG productivity. **l** Astaxanthin productivity. The values in **c**–**l** were recorded from day 4. The data are expressed as mean ± SD (*n* = 3). Different letters above the bars in each panel indicate significant difference (p < 0.05), based on one-way ANOVA with post hoc Tukey’s HSD test

To investigate the effect of salinity levels on growth and production of lipids and astaxanthin, sodium chloride (NaCl) was employed with five concentrations (0, 0.1, 0.2, 0.4, and 0.6 M). Apparently, the algal growth was impaired by salt treatment in a concentration-dependent manner: the higher the salt concentration, the lower the cell density and *F*v/*F*m value (Fig. 1a, b). Specifically, the growth was attenuated moderately by 0.1 and 0.2 M salt; by contrast, the alga showed nearly blocked proliferation in the presence of 0.4 and 0.6 M salt. Accordingly, the biomass concentration achieved after 4 days of cultivation was negatively associated with the salt concentration (Fig. 1c). Salt treatment also affected cell size as suggested by the elevated per cell weight (Fig. 1d). The salt-caused growth impairment to different degrees has been reported for many other freshwater algal strains [25–29, 31–35].

The content of total fatty acids (TFA) showed a considerable increase (over twofold) under the moderate salt concentration of 0.2 M, but did not increase further under high salt concentrations (Fig. 1e). Oleic acid (C18:1) that is believed to be beneficial to the quality of biodiesel [36], also increased in the presence of salt (Fig. 1f). Triacylglycerol (TAG), the most energy-dense lipid ideal for biodiesel production [1], exhibited a more drastic increase upon salt stress than TFA did and reached the highest content of 152 mg g^−1^ (ca. threefold increase) under 0.2 M salt concentration (Fig. 1g). On the other hand, the secondary ketocarotenoid astaxanthin showed a considerable increase under higher salt concentrations (0.2–0.6 M) and its content reached the maximum under 0.4 M salt concentration (3.1 mg g^−1^), which is 6.4-fold higher than that without salt treatment (Fig. 1h). From the production point of view, productivity is a more desirable parameter. Although the biomass productivity was attenuated by salt treatment (Fig. 1i), the productivities of TFA, TAG and astaxanthin were all promoted and reached the maximum under the moderate salt concentration of 0.2 M (Fig. 1j–l). These results together indicated that salt treatment is a simple and feasible strategy to boost both TAG and astaxanthin production, which is not only biotechnologically favorable, but also environmentally friendly, as it can reduce the usage of freshwater.

### Time-resolved biochemical analysis of *C. zofingiensis* in response to salt treatment

**Fig. 2:**
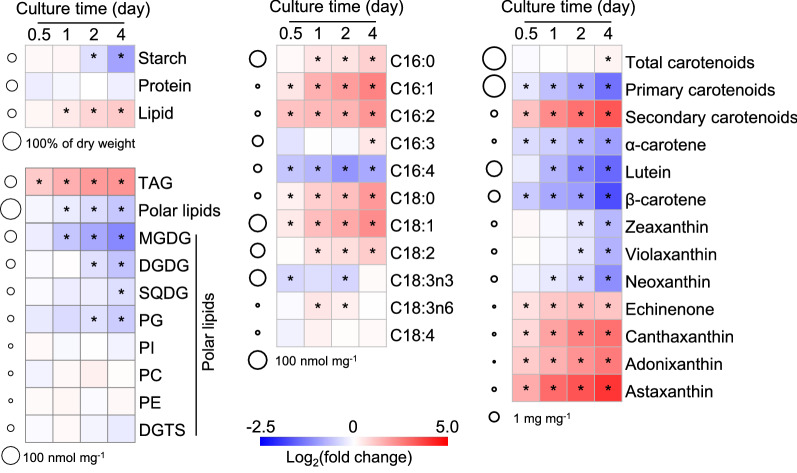
Heat map illustrating the variation of cellular content of major compounds including protein, starch, lipids and carotenoids. The changes in the compound contents in response to 0.2 M NaCl are expressed as log_2_(fold change) values (relative to day 0) and displayed in the heat map. Time refers to the duration (h) upon NaCl treatment. The circles at the left of the heat map designate the contents of compounds on day 0. The data are expressed as mean ± SD (*n* = 3). Significant difference (Student’s *t*-test, p < 0.05) is indicated with an asterisk

As demonstrated above, 0.2 M NaCl was the ideal salt concentration for both TAG and astaxanthin accumulation (Fig. 1). Under this concentration, the biochemical analysis was further investigated in a time-resolved manner to probe the dynamic changes of cellular compounds. Upon the salt treatment, protein showed little change (Fig. 2). This is different from nitrogen starvation (ND) treatment, where protein exhibited an immediate decrease [15]. Starch, the major carbohydrate reserve, maintained its content during the early treatment and decreased thereafter (Fig. 2). On the other hand, the total lipid content showed a gradual increase in response to salt treatment (Fig. 2). Specifically, the neutral lipid TAG had a basal level and was induced sharply by salt, accompanied by the decrease of polar lipids. A total of eight polar lipid classes were determined, namely, monogalactosyl diacylglycerol (MGDG), digalactosyl diacylglycerol (DGDG), sulfoquinovosyl diacylglycerol (SQDG), diacylglycerol-*N*,*N*,*N*-trimethylhomoserine (DGTS), phosphatidylglycerol (PG), phosphatidylcholine (PC), phosphatidylinositol (PI), and phosphatidylethanolamine (PE). In response to salt treatment, MGDG, the main component of thylakoid membrane, was severely attenuated; DGDG, SQDG, and PG were also decreased, but occurring during the late stress period to a lesser extent; by contrast, the others showed no significant change during the whole period (Fig. 2). The content of individual fatty acids was altered by salt treatment and most of them increased, though to various extents. Notably, C18:1, the major fatty acid found in *C. zofingiensis*, exhibited the greatest increase. Furthermore, taking into account the relative abundance of fatty acids, while saturated fatty acids (SFA) showed little change, monounsaturated fatty acids (MUFA) particularly C18:1 increased considerably at the expense of polyunsaturated fatty acids (PUFA) (Additional file 1: Figure S1).

Upon salt treatment, total carotenoids showed little variation until the late culture period (96 h), where a slight increase was observed. Specifically, there was a considerable decrease for primary carotenoids, particular β-carotene and lutein; by contrast, secondary carotenoids exhibited a vast increase, with astaxanthin and canthaxanthin being the most elevated ones. In this context, it is likely that secondary carotenoids accumulated at the expense of primary carotenoids, as suggested by the strong negative correlation (Additional file 1: Figure S2). It is worth noting that astaxanthin was present predominantly in the form of ester (both mono-ester and di-ester), which accounted for more than 80% of total astaxanthin (Additional file 1: Figure S3). Apparently, astaxanthin correlated well with TAG in response to salt treatment (Additional file 1: Figure S2), which is consistent with the results under other induction conditions [11] and further implies the coordinated biosynthesis of TAG and astaxanthin in *C. zofingiensis*.

In addition, the algal samples from 0 and 12 h of salt treatment were subjected to untargeted metabolomics analysis, which identified a total of 141 metabolites (Additional file 2: Data S1). Upon salt treatment, 50 metabolites (34%) underwent a significant change (at least a 1.5-fold change and p < 0.05): 38 decreased and 12 increased. The former metabolites were enriched in amino acid metabolism and TCA cycle, while the latter ones showed no specific enrichment.

### Transcriptomic analysis for global gene expression upon salt induction

**Fig. 3:**
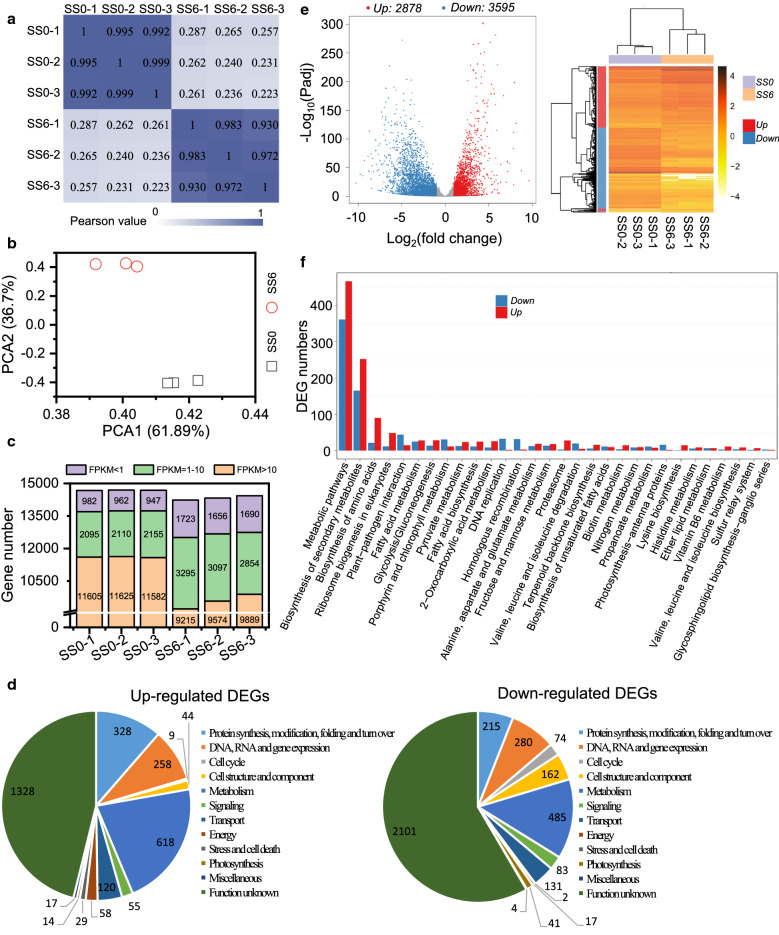
Global analysis of transcriptomes and DEGs. **a** Pearson correlation coefficients for transcriptomes between each two samples. **b** Principal component analysis (PCA) of the six transcriptomes. *X* and *Y* axes represent the contributor rate of first and second component, respectively. **c** The distribution of gene expression levels in each sample. **d** Volcano plot of DEGs. Red, blue and gray points represent up-regulated, down-regulated, and non-regulated DEGs, respectively. **e** Heat map showing the expression level (log_10_ transformed FPKM value) of DEGs in different samples. **f** KEGG pathway functional enrichment result for DEGs. Red and blue columns represent up-regulated and down-regulated DEGs, respectively. **g** Functional categories of DEGs with manual curation

To understand the underlying mechanisms of lipogenesis and carotenogenesis induced by salt stress, the global gene expression was investigated by RNA-seq in parallel with the biochemical analysis in *C. zofingiensis*. The algal samples from 0 and 6 h were used for RNA-seq, with biological triplicates for each time point. Six high-quality transcriptomes were produced (Additional file 3: Table S1): the biological triplicates in each group had a high consistence and distinguished from the other group based on the Pearson correlation and principal component analysis (Fig. 3a, b). Furthermore, quantitative real-time PCR (qPCR) was employed to validate the transcriptomes by analyzing 24 genes (Additional file 3: Table S2), and the plotting results indicated a high coefficient of 0.88 (*R*^2^) (Additional file 1: Figure S4). More than 14,000 genes were mapped for each sample, most of which had a FPKM (fragments per kilobase of transcript per million mapped reads) value no less than 1 (Fig. 3c and Additional file 4: Data S2).

Based on the definition of differentially expressed gene (DEG) stated in “Methods”, 6473 genes were assigned as salt-responsive DEGs (Additional file 5: Data S3): 2878 genes were up-regulated and 3595 were down-regulated (Fig. 3d, e). KEGG pathway functional enrichment analysis indicated several up-regulated (amino acid biosynthesis, ribosome biogenesis, glycolysis, proteasome-related, etc.) and down-regulated (chlorophyll metabolism, DNA replication, photosynthesis-antenna proteins, etc.) pathways in response to salt stress (Fig. 3f; Additional file 6: Data S4). According to Liu et al. [15], the DEGs were also manually categorized into 12 groups (Fig. 3g; Additional file 7: Data S5). The genes in function unknown category have the greatest percentage for both up-regulated (46%) and down-regulated (58%) DEGs. This is not surprising as about half of the genes in *C. zofingiensis* were annotated with unknown function [18]. Metabolism represents the second largest category for both up-regulated and down-regulated DEGs, suggesting the complex regulation of metabolic pathways upon salt treatment. In the categories of photosynthesis, cell cycle, and cell structure and component, there are much more down-regulated DEGs than up-regulated DEGs, indicative of the repression of photosynthesis and cell division, which is consistent with the retarded cell proliferation caused by salt treatment (Fig. 1).

Furthermore, considering the particular interest of this study in lipid metabolism and carotenoid synthesis, all putative genes involved in the pathways were manually identified and their expression profiles were listed in Additional file 8: Data S6 and Additional file 9: Data S7, respectively. Of the 192 lipid metabolism-related genes, 94 were DEGs: 61 (65%) were up-regulated and 33 (35%) were down-regulated. Of the 35 carotenoid synthesis-related genes, 16 belonged to DEGs. The regulation of lipid metabolism and carotenoid synthesis in response to salt stress are detailed in the subsequent sections by the congruent analysis of RNA-seq data and biochemical variations.

### Salt stress promotes fatty acid synthesis while attenuating its β-oxidation

**Fig. 4:**
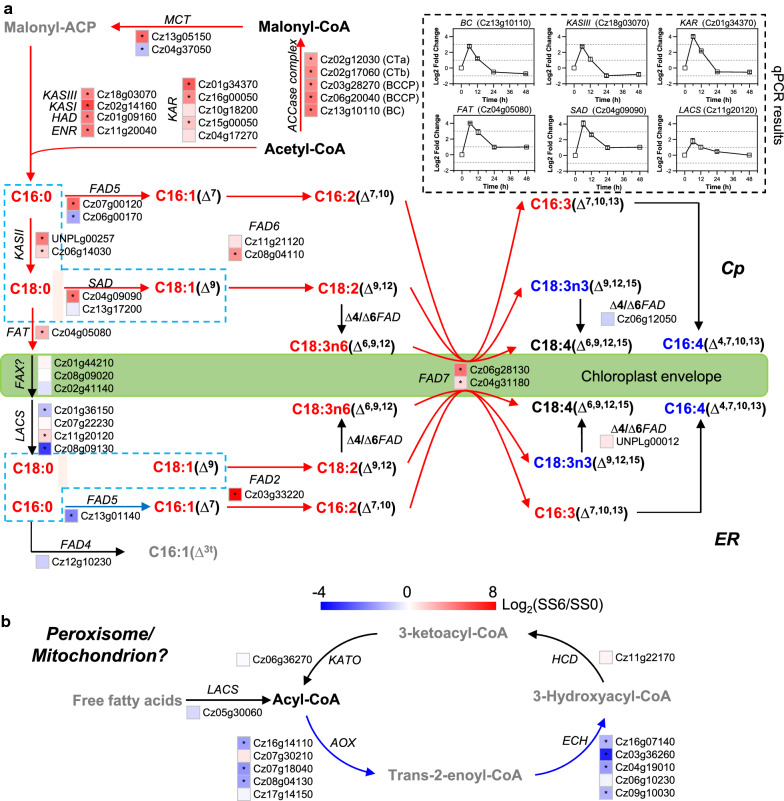
Regulation of fatty acid biosynthesis (**a**) and β-oxidation (**b**) in response to salt stress. The heat map right before gene IDs illustrates gene expression changes (log_2_ transformed values). Significant difference (at least a twofold change and FDR adjusted *p *< 0.05) is indicated with an asterisk. qPCR was employed to exam the time-resolved expression of five selected genes and the results are shown at the right up corner. Arrows in red, blue, and black designate the up-regulated, down-regulated, and no-regulated steps, respectively. For proteins encoded by multiple gene copies, the changes in total transcripts of the isogenes were employed for determining the overall regulation pattern. Compounds are highlighted with different colors: red, significantly higher; black, not significantly changed; gray, not determined; blue, significantly lower upon salt stress. *ACCase* acetyl-CoA carboxylase, *AOX* acyl-CoA oxidase, *BC* biotin carboxylase, *BCCP* biotin carboxyl carrier protein, *CT* carboxyltransferase, *ECH* enoyl-CoA hydratase, *HCD* 3-hydroxyacyl-CoA dehydrogenase, *ENR* enoyl-ACP reductase, *FAD* fatty acid desaturase, *FAT* acyl-ACP thioesterase, *HAD* 3-ketoacyl-ACP dehydratase, *KAR* 3-ketoacyl-ACP reductase, *KAS* 3-ketoacyl-ACP synthase, *KATO* 3-ketoacyl-CoA thiolase, *LACS* long-chain acyl-CoA synthetase, *MCT* malonyl-CoA:acyl carrier protein transacylase, *SAD* stearoyl-ACP desaturase. See Additional file 8: Data S6 for the detailed RNA-seq data

Resembling higher plants, the de novo fatty acid synthesis in algae occurs in the chloroplast and involves a set of enzymes [37]. Using acetyl-CoA as the substrate, acetyl-CoA carboxylase (ACCase) catalyzes the formation of malonyl-CoA, which is regarded as the first committed step for the de novo fatty acid synthesis [38]. Searching *C. zofingiensis* genome identified a prokaryotic form of ACCase consisting of four chloroplast-targeted subunits: carboxyltransferase subunits alpha (Cz02g12030) and beta (Cz02g17060), biotin carboxyl carrier protein (two isoforms, Cz03g28270 and Cz06g20040), and biotin carboxylase (Cz13g10110), and a eukaryotic multifunctional form (Cz19g10190) targeted to the cytosol (Fig. 4a; Additional file 8: Data S6). In response to salt stress, all subunits of the chloroplastic ACCase were considerably up-regulated (over eightfold), suggesting that the chloroplastic ACCase represents a committed enzyme controlling the biosynthesis of fatty acids in *C. zofingiensis*. The salt-induced expression of ACCase has also been reported in some other algae including *Chlamydomonas* [26, 39, 40], *Chlorella* [41], and *Nitzschia* [42]. Obviously, in *C. zofingiensis* the transcriptional expression of ACCase subunits correlated well with each other (Additional file 8: Data S6), supporting their coordinated regulation as stated by previous reports [43, 44]. The cytosolic ACCase, on the other hand, was down-regulated considerably by salt stress. The malonyl moiety of malonyl-CoA is transferred to an acyl carrier protein (ACP) by the action of a malonyl-CoA:acyl carrier protein transacylase (MCT) leading to the formation of malonyl-CoA for the subsequent condensation reactions to form C16 and/or C18 fatty acids. Both *ACP* and *MCT* genes were considerably up-regulated by salt stress (Fig. 4a; Additional file 8: Data S6). However, malonyl-CoA, the product of MCT, showed no change in response to the stress (Additional file 2: Data S1). This may be attributed to the considerable up-regulation of downstream fatty acid synthesis genes (Fig. 4a), which allows rapid consumption of malonyl-CoA and thus the maintaining of its homeostasis.

Fatty acid synthesis from malonyl-ACP involves a set of fatty acid synthases consisting of 3-ketoacyl-ACP synthase (KAS), 3-ketoacyl-ACP reductase (KAR), 3-hydroxyacyl-ACP dehydratase (HAD), and enoyl-ACP reductase (ENR) [38]. In *C. zofingiensis*, four *KAS*, five *KAR*, one *HAD*, and one *ENR* genes were found (Fig. 4a; Additional file 8: Data S6). Three types of KAS are present in higher plants responsible for the condensation of acyl-ACP with acetyl-CoA: KAS III catalyzes the first condensation of malonyl-ACP and acetyl-CoA to form C4:0-ACP, KAS I catalyzes the subsequent condensation reactions up to the formation of C16:0-ACP, while KAS II catalyzes the formation of C18:0-ACP from C16:0-ACP, the last condensation step in the chloroplast. Both *C. zofingiensis* KAS III and KAS I have a single-copy gene encoding a chloroplastic form (Cz18g03070 and Cz02g14160); by contrast, KAS II have two genes, one (UNPLg00257) for a chloroplastic form and the other (Cz06g14030) for a mitochondrial form: all three chloroplastic genes were greatly up-regulated (over tenfold) upon salt stress. Of the five *KAR* genes, only one (Cz01g34370) encoded a chloroplastic form, which together with *HAD* (Cz01g09160) and *ENR* (Cz11g20040) had comparable transcript levels and were up-regulated to similar extent. Furthermore, *BC*, *KAS III*, and the chloroplastic *KAR* genes were chosen for time-resolved qPCR analysis to validate gene expression profiles. All three genes showed a dramatic increase and reached their maximum after 6 h of salt stress, highly consistent with the RNA-seq data (Fig. 4a). Taken together, salt stress strongly stimulated the entire pathway for de novo fatty acid synthesis in a well-coordinated manner, thus allowing the effective utilization of acetyl-CoA to accumulate C16:0, C18:0, and C18:1 in *C. zofingiensis* (Fig. 4a).

The de novo synthesized fatty acids (in the form of acyl-ACP) can be either directly incorporated into glycerolipids by chloroplast-localized acyltransferases or released as free fatty acids by an acyl-ACP thioesterase (FAT) [37]. A single-copy *FAT* gene (Cz04g05080) was identified in *C. zofingiensis* and its transcript was up-regulated considerably based on both RNA-seq and qPCR results (Fig. 4a). The released free fatty acids need to be exported across chloroplast envelopes, which is mediated by a fatty acid export 1 (FAX1) in Arabidopsis [45], and ligated to CoA via a long-chain acyl-CoA synthetase (LCAS), prior to utilization by the ER-localized acyltransferases for glycerolipid assembly. The FAX1 homologues in *C. zofingiensis* showed little variations upon salt stress (Fig. 4a). Among the four identified putative *LCAS* genes, all had a similar level of basal transcripts but only Cz11g20120 was up-regulated by salt stress (Fig. 4a; Additional file 8: Data S6). Similar to *C. reinhardtii* LCAS2 which contributes to TAG accumulation [46], Cz11g20120 may play a critical role in salt-induced TAG accumulation in *C. zofingiensis*.

The unsaturation degree of fatty acids is determined by a series of desaturases via an oxygen-dependent mechanism [37, 38]. Fatty acid desaturases are usually membrane-bound enzymes utilizing complex lipids as substrates [37]. An exception is the stearoyl-ACP desaturase (SAD), a chloroplast stroma-localized soluble enzyme catalyzing the insertion of a *cis* double bond to the ∆9 position of C18:0-ACP to form C18:1-ACP [47, 48]. Two *SAD* genes were found in *C. zofingiensis*: Cz04g09090 was considerably up-regulated (both RNA-seq and qPCR results), while Cz13g17200 had no change upon salt stress (Fig. 4a; Additional file 8: Data S6). Thus, it is likely the Cz04g09090 rather than Cz13g17200 that contributes to the tremendous increase in the cellular content of C18:1 (Fig. 2). Other identified desaturase genes included *FAD2* (Cz03g33220), *FAD4* (Cz12g10230), *FAD5* (Cz07g00120, Cz06g00170, and Cz13g01140), *FAD6* (Cz08g04110 and Cz11g21120), *FAD7* (Cz06g28130 and Cz04g31180), and ∆*4/*∆*6FAD* (Cz06g12050 and UNPLg00012) (Fig. 4a; Additional file 8: Data S6). FAD2 is an ER-targeted desaturase responsible for introducing a double bond to the ∆12 position of C18:1 to form C18:2 bound to extrachloroplastic membrane lipids such as PE, PC, PI, and DGTS. By contrast, FAD6 is localized in the chloroplast and catalyzes the formation of C18:2 from C18:1 in the chloroplastic membrane lipids, e.g., MGDG, DGDG, SQDG, and PG. FAD7, on the other hand, resides likely in the chloroplast envelop and accesses both extrachloroplastic and chloroplastic lipids for the desaturation of C18:2 at the ∆15 position to form C18:3 [37, 49]. In *C. zofingiensis*, most *FAD* genes were up-regulated by salt stress (Fig. 4a; Additional file 8: Data S6), leading to enhanced cellular contents of unsaturated fatty acids (Fig. 2).

Fatty acids can enter β-oxidation pathway for degradation, which involves a set of enzymes including LACS, acyl-CoA oxidase (AOX), enoyl-CoA hydratase (ECH), 3-hydroxyacyl-CoA dehydrogenase (HCD) and 3-ketoacyl-CoA thiolase (KATO) [37]. Overall, *AOX* and *ECH* genes were down-regulated (Fig. 4b; Additional file 8: Data S6), indicative of attenuated β-oxidation of fatty acids under salt stress conditions.

### Salt stress likely stimulates the turnover of membrane lipids

**Fig. 5:**
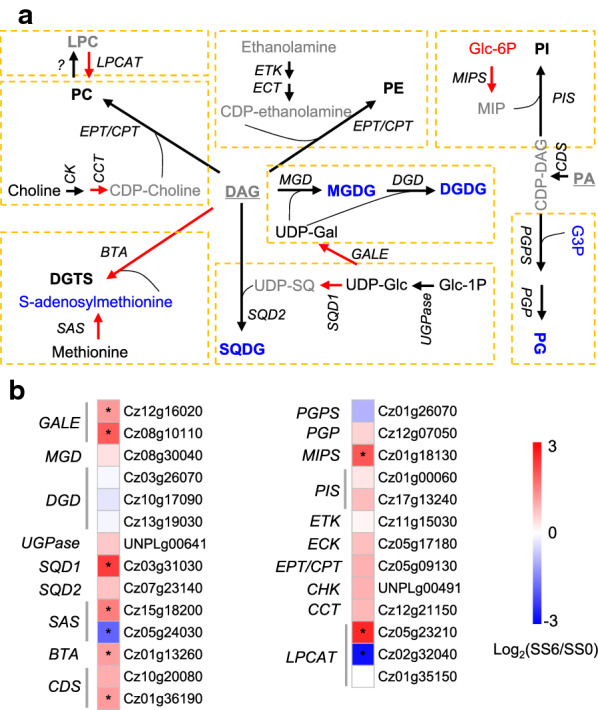
Regulation of membrane glycerolipid synthesis in response to salt stress. **a** Transcriptional regulation of membrane lipid biosynthetic pathways. Arrows in red and black indicate transcriptional up- and non-regulated steps, respectively. For proteins encoded by multiple gene copies, the changes in total transcripts of the isogenes were employed for determining the overall regulation pattern. Compounds are highlighted with different colors: red, significantly higher; black, not significantly changed; gray, not determined; blue, significantly lower upon salt stress. **b** Heat map showing log_2_(fold change) values of gene transcripts. Significant difference (at least a twofold change and FDR adjusted p < 0.05) is indicated with an asterisk. *BAT* betaine lipid synthase, *CDS* phosphatidate cytidylyltransferase, *CCT* choline-phosphate cytidylyltransferase, *CHK* choline kinase, *DGD* digalactosyldiacylglycerol synthase, *DGDG* digalactosyl diacylglycerol, *ECT* CDP-ethanolamine synthase, *EPT/CPT* ethanolaminephosphotransferase/cholinephosphotransferase, *ETK* ethanolamine kinase, *GALE* UDP-galactose 4-epimerase, *LPC* lysophosphatidylcholine, *MIPS* myo-inositol-1-phosphate synthase, *MGD* monogalactosyldiacylglycerol synthase, *MGDG* monogalactosyl diacylglycerol, *PA* phosphatidic acid, *PC* phosphatidylcholine, *PE* phosphatidylethanolamine, *PG* phosphatidylglycerol, *PGP* phosphatidylglycerophosphatase, *PGPS* phosphatidylglycerophosphate synthase, *PI* phosphatidylinositol, *PIS* phosphatidylinositol synthase, *UGPase* UDP-glucose pyrophosphorylase, *SAS S*-adenosylmethionine synthase, *SQDG* sulfoquinovosyl diacylglycerol. See Additional file 8: Data S6 for the detailed RNA-seq data

It has been reported in algae that membrane lipids undergo turnover to provide fatty acyls for TAG assembly under abiotic stress conditions such as nitrogen deficiency (ND) [15, 50–54]. Similarly, salt stress also caused a decrease in polar lipids, but predominantly in the chloroplastic membrane lipids (MGDG, DGDG, SQDG and PG) (Figs. 2, 5a); this differs from the changes under ND conditions where all determined polar lipids were decreased [15]. However, the biosynthesis of polar lipids were not down-regulated at the transcriptional level; instead, some genes were even up-regulated by salt stress (Fig. 5b; Additional file 8: Data S6). It is likely that upon salt treatment *C. zofingiensis* maintained the synthesis of membrane glycerolipids, but up-regulated the expression of lipases responsible for membrane lipid degradation, thereby stimulating the turnover towards decreased polar lipids. There are a number of putative membrane lipase-encoding genes found in algae and many were considerably up-regulated under TAG induction conditions [15, 55–57]. Among these lipases, PGD1 from *C. reinhardtii* has been well investigated and showed to play a role in MGDG degradation [52]. Upon salt treatment, the PGD1 homologue in *C. zofingiensis* also showed a large up-regulation (Additional file 8: Data S6), suggesting its role in MGDG turnover. There are additional lipase genes that were up-regulated, such as Cz09g17170, Cz14g02110 and Cz19g09050 (Additional file 8: Data S6). They may be involved in the turnover of other membrane lipids in *C. zofingiensis* to provide fatty acids for TAG synthesis.

### Salt stress induces the expression of TAG assembly and lipid droplet proteins for TAG accumulation

TAG assembly in algae is thought to be mediated mainly by acyl-CoA-dependent Kennedy pathway and acyl-CoA-independent pathway [37, 58]. It is thought in *C. reinhardtii* that the former pathway contributes more than the latter one to abiotic stress-associated TAG synthesis [53, 59]. The acyl-CoA-dependent Kennedy pathway starts with glycerol-3-phosphate and involves a set of enzymes including glycerol-3-phosphate acyltransferase (GPAT), lysophosphatidic acid acyltransferase (LPAAT), phosphatidate phosphatase (PAP) and diacylglycerol acyltransferase (DGAT). *C. zofingiensis* possesses two *GPAT*s, three *LPAAT*s, three *PAP*s and ten *DGAT*s (Fig. 5a; Additional file 8: Data S6). Upon salt stress, *GPAT2* and *LPAAT1* were up-regulated (Fig. 5a, b), consistent in principle with the results under ND conditions [15], supporting the role of the two acyltransferases in TAG synthesis under different abiotic stress conditions. Interestingly, under salt stress conditions, *PAP3* rather than *PAP1* was up-regulated (Fig. 5a, 5b), while under ND conditions, *PAP1* instead of *PAP3* was up-regulated [15]. This suggests that *C. zofingiensis* may adopt different PAPs to cope with salt and ND stresses for TAG assembly.

DGAT catalyzes the last committed step and has been demonstrated to play a critical role in TAG accumulation in various algal species [53, 54, 60–62]. Recently, we showed by functional complementation in TAG-deficient yeast that seven out of the ten *C. zofingiensis DGAT*s were able to restore TAG synthesis, with *DGAT1*A being most functional followed by *DGTT5* and *DGAT1B* [63]. Upon salt treatment, only *DGTT5* was up-regulated (Fig. 5a, 5b), indicative of its critical role in salt-induced TAG accumulation in *C. zofingiensis*. By contrast, seven *DGAT* genes including *DGAT1A* and *DGTT5* were up-regulated under ND conditions [15, 63]. These may partially explain why salt stress induced less TAG than ND did in *C. zofingiensis* (Fig. 1g; [63]). Furthermore, DGAT1A preferred eukaryotic DAGs (sn-2 being C18:1) while DGTT5 preferred prokaryotic DAGs (sn-2 being C16:0) [63]. In consistence, salt-induced TAG had a lower percentage of eukaryotic TAG than ND-induced TAG (Additional file 1: Figure S5).

The acyl-CoA-independent pathway for TAG synthesis is catalyzed by phospholipid:diacylglycerol acyltransferase (PDAT), which transfers an acyl moiety from phospholipids and/or other polar lipids to DAG for TAG synthesis [59]. *C. zofingiensis PDAT* was up-regulated upon salt stress, to the same extent as *DGTT5* (Fig. 5a; Additional file 8: Data S6). Therefore, PDAT may also contribute essentially to TAG accumulation under salt conditions. It has been reported recently that PDAT interacts with DGAT for TAG assembly in higher plants [64]. This interaction may also occur for PDAT and DGTT5 in *C. zofingiensis*.

**Fig. 6:**
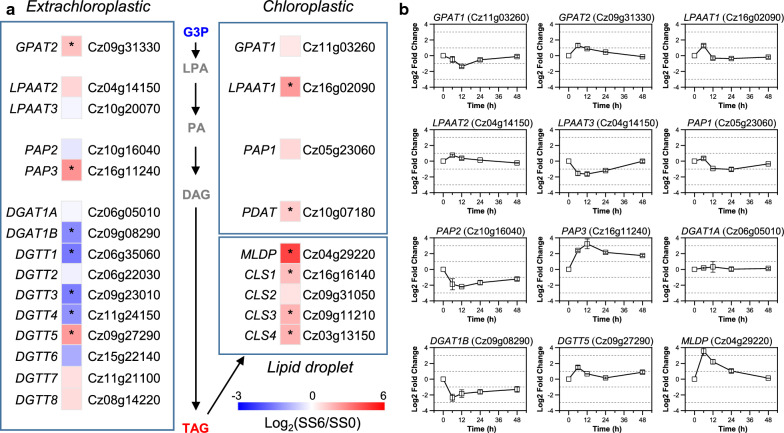
Regulation of TAG assembly in response to salt stress. **a** Transcriptional regulation of TAG assembly pathways. A heat map showing log_2_(fold change) values of gene transcripts. Significant difference (at least a twofold change and FDR adjusted p < 0.05) is indicated with an asterisk. Compounds are highlighted with different colors: red, significantly higher; black, not significantly changed; gray, not determined; blue, significantly lower upon salt stress. **b** Time-resolved expression of selected genes determined by qPCR. *CLS* caleosin, *DAG* diacylglycerol, *DGAT* Diacylglycerol acyltransferase, *DGAT1* type I DGAT, DGTT type II DGAT, *G3P* glycerol-3-phosphate, *GPAT* glycerol-3-phosphate acyltransferase, *LPA* lysophosphatidic acid, *LPAAT* lysophosphatidic acid acyltransferase, *MLDP* major lipid droplet protein, *PAP* phosphatidate phosphatase, *PDAT* phospholipid:diacylglycerol acyltransferase, *TAG* triacyglycerol. See Additional file 8: Data S6 for the detailed RNA-seq data

TAG, once synthesized, is packed into lipid droplets (LDs), the lipid-rich cellular organelles that regulate the storage and hydrolysis of neutral lipids [65]. LDs occur not only in cytosol, but also within chloroplast, which has been demonstrated in *Chlamydomonas* [66, 67]. Unlike *Chlamydomonas*, *C. zofingiensis* forms only cytoplasmic LDs, which are stimulated to grow in size under ND conditions [16]. The stabilization of LDs involves certain structural proteins, such as oleosin in *Arabidopsis* [68], MLDP in *Chlamydomonas* [69, 70] and *Dunaliella* [71], HOGP in *Haematococcus* [72], LDSP in *Nannochloropsis* [73], and StLDP in *Phaeodactylum* [74]. In *C. zofingiensis*, Cz04g29220, encoding a homologue to MLDP of green algal lineage, had a high transcript level (FPKM = 199) under non-stress conditions and exhibited a considerable up-regulation (~ 17-fold) upon salt stress (Additional file 8: Data S6). By contrast, *Chlamydomonas* had a much lower basal transcript level of MLDP [55], in correlation with the fact that under favorable conditions, *Chlamydomonas* contained trace amounts of TAG with no detectable LDs [53, 66], while *C. zofingiensis* accumulated TAG ~ 5% of dry weight with visible LDs peripherally scattered (Fig. 1; [16]). In addition to MLDP, *C. zofingiensis* harbored several caleosin genes (Cz16g16140, Cz09g31050, Cz09g11210 and Cz03g13150), which were up-regulated by salt stress (Fig. 6a; Additional file 8: Data S6). Unlike MLDP that lacks a specific hydrophobic core characteristic, caleosin has hydrophobic segments and a Ca^2+^-binding motif, possesses peroxygenase activities and is believed to be involved in oxylipin synthesis for combating stresses [75]. We recently demonstrated that *C. zofingiensis* MLDP and caleosins were localized in LDs with a comparable abundance [76]. The up-regulation of both MLDP and caleosins likely guarantee the stabilization of LDs as the sink for TAG storage and protect TAG against degradation under salt stress conditions.

### Salt stress enhances astaxanthin accumulation at the expense of primary carotenoids

**Fig. 7:**
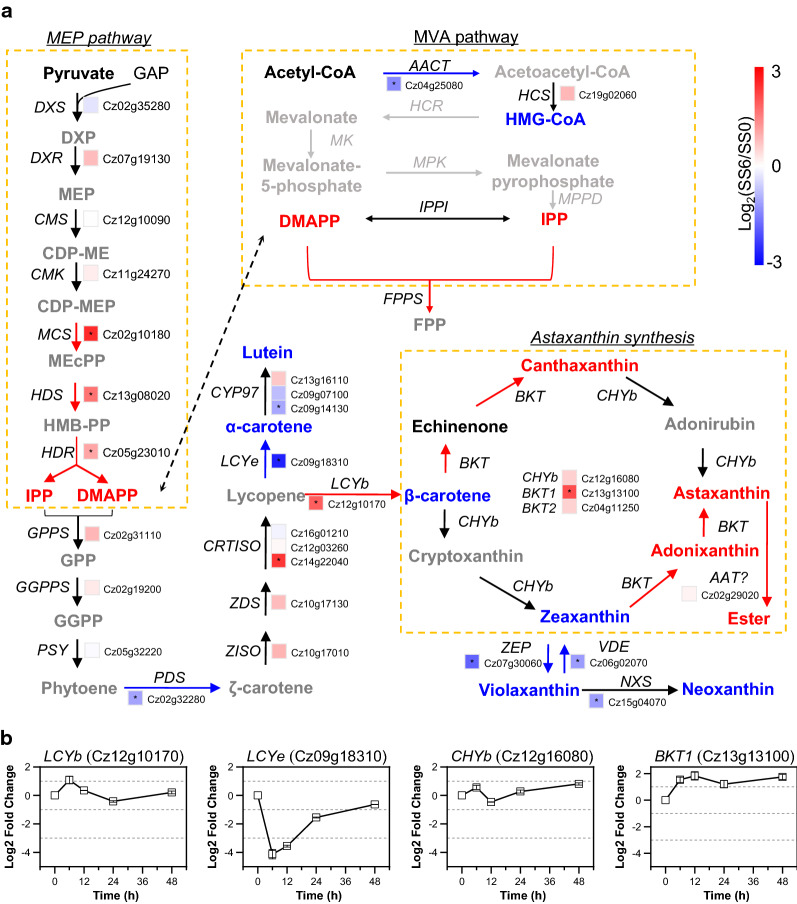
Regulation of carotenogenesis in response to salt stress. **a** Transcriptional regulation of carotenogenesis. The heat map right before gene IDs illustrates gene expression changes (log2 transformed values). Significant difference (at least a twofold change and FDR adjusted *p *< 0.05) is indicated with an asterisk. Arrows in red, blue, and black indicate transcriptional up-, down-, and non-regulated steps, respectively. For proteins encoded by multiple copies of genes, the changes in total transcripts of the isogenes were employed for determining the overall regulation pattern. Compounds are highlighted with different colors: red, significantly higher; black, not significantly changed; gray not determined; blue, significantly lower under upon salt stress. **b** Time-resolved expression of selected genes determined by qPCR. *AACT* acetoacetyl-CoA thiolase, *AAT* long-chain-alcohol *O*-fatty-acyltransferase, *BKT* beta-carotenoid ketolase, *CDP-ME* 4-diphosphocytidyl-2-C-methylerythritol, *CDP-MEP* 4-diphosphocytidyl-2-C-methyl-d-erythritol 2-phosphate, *CHYb* beta-carotenoid hydroxylase, CMK 4-diphosphocytidyl-2-C-methyl-d-erythritol kinase, *CMS* 2-C-methyl-d-erythritol 4-phosphate cytidylyltransferase, *CRTISO* carotenoid isomerase, *CYP97A* cytochrome P450 beta hydroxylase, *CYP97C* cytochrome P450 epsilon hydroxylase, *DMAPP* dimethylallyl pyrophosphate, *DXR* 1-deoxy-d-xylulose 5-phosphate reductoisomerase, *DXP* 1-deoxy-d-xylulose 5-phosphate, *DXS* 1-deoxy-d-xylulose 5-phosphate synthase, *FPP* farnesyl diphosphate, *FPPS* farnesyl diphosphate synthase, *GAP* glyceraldehyde 3-phosphate, *GGPP* geranylgeranyl diphosphate, *GGPPS* geranylgeranyl diphosphate synthase, *GPP* geranyl diphosphate, *GPPS* geranyl diphosphate synthase, *HCS* hydroxymethylglutaryl-CoA synthase, *HDR* 4-hydroxy-3-methylbut-2-en-1-yl diphosphate reductase, *HDS* 4-hydroxy-3-methylbut-2-en-1-yl diphosphate synthase, *HGM-CoA* 3-hydroxy-3-methylglutaryl-CoA, *HMB-PP* (E)-4-Hydroxy-3-methyl-but-2-enyl pyrophosphate, *IPP* isopentenyl pyrophosphate, *IPPI* isopentenyl-diphosphate Delta-isomerase, *LCYb* lycopene beta cyclase, *LCYe* lycopene epsilon cyclase, *GPP* geranyl diphosphate, MCS 2-C-methyl-d-erythritol 2,4-cyclodiphosphate synthase, *MEcPP* 2-C-methyl-d-erythritol 2,4-cyclodiphosphate, *MEP* 2-C-methylerythritol 4-phosphate, *NXS* neoxanthin synthase, *PDS* phytoene desaturase, *PSY* phytoene synthase, *VDE* violaxanthin de-epoxidase, *ZDS* zeta-carotene desaturase, ZEP zeaxanthin epoxidase, *ZISO* zeta-carotene isomerase. See Additional file 9: Data S7 for the detailed RNA-seq data

It has been suggested in green algae that the biosynthesis of carotenoids employs precursors derived from the chloroplastic methylerythritol phosphate (MEP) pathway rather than the cytosolic mevalonate (MVA) pathway [77–79]. In *C. zofingiensis*, all enzymes involved in the MEP pathway are present and appear to be encoded by single-copy genes, while some enzymes in the MVA pathway are missing (Additional file 9: Data S7), supporting that green algae may abandon the MVA pathway for supplying the building blocks for cellular isoprenoids [79]. The MEP pathway is initiated by 1-deoxy-d-xylulose 5-phosphate (DXP) synthase (DXS), which catalyzes the irreversible condensation of pyruvate and glyceraldehyde 3-phosphate (GAP) to form DXP. DXP is then converted to MEP mediated by DXP reductoisomerase (DXR), the first committed step of the MEP pathway towards isoprenoid synthesis. The last three steps involves 2-C-methyl-D-erythritol 2,4-cyclodiphosphate synthase (MCS), 4-hydroxy-3-methylbut-2-en-1-yl diphosphate synthase (HDS) and reductase (HDR), catalyzing the formation of 5-carbon isoprenoids isopentenyl pyrophosphate (IPP) and dimethylallyl pyrophosphate (DMAPP). In *C. zofingiensis* upon salt treatment, although the initial steps showed little changes, the last three steps were up-regulated (Fig. 7a; Additional file 9: Data S7), suggesting the stimulation of the MEP pathway.

IPP and DMAPP, the building blocks of carotenoids, are converted to the 10-carbon geranyl diphosphate (GPP), followed by the condensation of two molecules of GPP to form geranylgeranyl pyrophosphate (GGPP). GGPP is further condensed to the first 40-carbon carotene phytoene, mediated by phytoene synthase (PSY). The colorless phytoene, after several desaturation and isomerization steps, is converted to lycopene. Interestingly, the genes encoding the enzymes involved in the formation of lycopene from 5-carbon isoprenoids were not up-regulated by salt stress; conversely, phytoene desaturase (PDS), which showed an up-regulation upon high light [80], was even down-regulated (Fig. 7a). This, together with up-regulation of the MEP pathway, may explain partially the build-up of IPP and DMAPP under salt stress conditions (Fig. 7a; Additional file 2: Data S1). They may be exported out of the chloroplast and serve as the precursors for sterol synthesis [79].

Lycopene represents the branch point for α-carotene and β-carotene, which enter into the biosynthesis of lutein and astaxanthin, respectively. The severe down-regulation of lycopene ε-cyclase (LCYe, eightfold decrease) and up-regulation of lycopene β-cyclase (LCYb) likely divert the carotenoid flux away from lutein (Fig. 7a, b), thereby leading to the considerable decrease of lutein level under salt stress conditions (Fig. 2). Astaxanthin biosynthesis employs β-carotene as the direct precursor, involving multiple routes via a series of hydroxylation and ketolation steps mediated by β-carotene hydroxylase (CHYb) and ketolase (BKT). *C. zofingiensis* possesses one *CHYb* gene (Cz12g16080) and two *BKT* genes, *BKT1* (Cz13g13100) and *BKT2* (Cz04g11250). *CHYb* and *BKT1* have been characterized by functional complementation in pathway-reconstructed *E. coli* cells [81, 82]. Upon salt stress, *BKT1* rather than *BKT2* or *CHYb* was up-regulated (Fig. 7), suggesting its contribution to salt-induced astaxanthin accumulation. The up-regulation of *BKT1* has also been observed previously under various conditions [17, 18, 80, 82, 83], suggesting the critical role of BKT1 in astaxanthin biosynthesis regardless of induction conditions. This has been further confirmed recently through the characterization of *bkt1* mutants, in which astaxanthin was almost abolished [18, 84]. Collectively, salt stress stimulated astaxanthin biosynthesis, particularly *BKT1* while repressing lutein synthesis, therefore rerouting the carotenoid flux to accumulate secondary carotenoids including astaxanthin at the expense of primary carotenoids.

## Discussion

### Salt stress triggers a global response of *C. zofingiensis*

Salt stress is a well-known abiotic stress that has multiplex effects on photosynthetic eukaryotes, such as photosynthesis impairment, ROS accumulation, protein turnover, glycerol build-up, oil accumulation, or carotenogenesis, depending on organisms [9, 28, 29, 40, 85–87]. The adverse impact of salt stress on photosynthesis has been observed for freshwater algae particularly the model alga *Chlamydomonas reinhardtii*, leading to attenuated biomass production in response to salt stress [28, 40, 88]. In *C. zofingiensis*, the vast majority of genes involved in chlorophyll biosynthesis, photosystem I and II, cytochrome b6/f complex and light harvest complexes showed a severe down-regulation upon salt stress (Additional file 10: Data S8). This, together with the decrease in photosynthetic pigments (chlorophylls, β-carotene, lutein, etc.) and chloroplast membrane lipids (MGDG, DGDG, PG, and SQDG) (Fig. 2; Additional file 1: Figure S6), firmly supports the impairment of the light reactions of photosynthesis. Similarly to higher plants, the Calvin–Benson cycle is believed to play a major role in algae for the photosynthetic fixation of CO_2_ [89, 90]. Ribulose 1,5-bisphosphate carboxylase/oxygenase (Rubisco), which catalyzes the carboxylation of ribulose 1,5-bisphosphate (RuBP), is a highly conserved, rate-limiting enzyme that initiates the cycle [91]. The activity of Rubisco, on the other hand, is regulated by Rubisco activase in an ATP-dependent manner [92]. The regeneration of RuBP also plays an important role in controlling the Calvin–Benson cycle for CO_2_ fixation [93]. Upon salt stress, *C. zofingiensis* exhibited a considerable down-regulation for Rubisco (over 15-fold decrease), Rubisco activase and phosphoribulokinase (responsible for the last step of RuBP regeneration) at the transcriptional level (Additional file 11: Data S9); accordingly, a decrease was observed for 3-phosphoglycerate, the C3 product of carboxylation (Additional file 2: Data S1), indicative of the attenuation of Calvin–Benson cycle. In this case, upon salt treatment, the light reactions were severely repressed leading to less production of ATP and NADPH molecules for photosynthetic fixation of CO_2_ and therefore the attenuated CO_2_ fixation ability. As a consequence, *C. zofingiensis* had retarded cell growth and impaired biomass production (Fig. 1). This may also be a transient adaptive feature for survival as it allows algal cells to rely on more resources such as energy and building blocks to combat the salt stress.

Salt stress has been demonstrated to exaggerate the generation of intracellular ROS in higher plants and algae [29, 40, 94]. Similarly, a considerable increase in ROS level was observed in salt-treated *C. zofingiensis* cells [83]. While serving as secondary messengers at basal levels, ROS in excess induces oxidative stress and is harmful to organisms [94]. To cope with the adverse effect of excess ROS, phototrophs have developed complex antioxidant mechanisms mediated by enzymatic components and non-enzymatic antioxidants; the former include superoxide dismutase (SOD), catalase (CAT), glutathione peroxidase (GPX), glutathione reductase (GR) and ascorbate peroxidase (APX), while the latter are composed of ascorbic acid, reduced glutathione, proline, carotenoids, flavonoids, etc. [95]. Different algae may adopt various scavenging strategies for salt-induced ROS [28, 29, 40, 86]. In case of *C. zofingiensis*, upon salt stress, *SOD* genes were somewhat up-regulated, *GPX* genes were up-regulated, while *CAT* and *APX* genes were somewhat down-regulated (Additional file 12: Data S10). Therefore, SOD and GPX may contribute to enzymatic detoxification of ROS in *C. zofingiensis* under salt stress conditions. As for the non-enzymatic antioxidants, ascorbic acid, reduced glutathione, and proline that have been reported to accumulate upon salt treatment in other algae [28, 40], showed no increase in *C. zofingiensis* (Additional file 2: Data S1). By contrast, secondary carotenoids particularly astaxanthin were considerably promoted by salt stress (Fig. 2), pointing to the important role of astaxanthin in the non-enzymatic sequestration of ROS in *C. zofingiensis*, as is the case in *H. pluvialis* [96].

Different algae have different salt tolerance capacities [9, 28, 39, 40]. Here were found that *C. zofingiensis* could tolerate NaCl up to 0.2 mM; further increase of salt concentration, however, nearly blocked the cell growth (Fig. 1). The adverse effect on algae imposed by salt stress may lie in the build-up of the excessive sodium ions in the cells and the hyperosmotic stress. Thus, organisms need to maintain both ionic and osmotic homeostasis [97]. Higher plants have evolved two main strategies to alleviate the build-up of Na^+^ in cytosol: efflux out of cells mediated by a plasma-membrane Na^+^/H^+^ antiporter encoded by the *SOS1* gene and sequestration into vacuole by a vacuolar Na^+^/H^+^ exchanger encoded by *NHX* gene [98]. The corresponding genes in *C. zofingiensis*, however, showed no up-regulation in response to salt stress (Additional file 12: Data S10). In this case, the alga may adopt different mechanisms for Na^+^ detoxification. The synthesis and accumulation of glycerol is a strategy for cells to combat osmotic pressure, which has been observed in *C. reinhardtii* in response to salt stress [99]. In *C. zofingiensis*, glycerol-3-phosphatase (GPP) was up-regulated upon salt stress (Additional file 13: Data S11), but not upon ND [15], indicating that glycerol production might be stimulated under salt stress conditions.

Nitrogen metabolism was also affected severely by salt stress in *C. zofingiensis*. The transport and assimilation of various nitrogen sources including urea, nitrate, nitrite and ammonia were stimulated in response to salt stress (Additional file 14: Data S12). This may provide enough nitrogen sources for amino acid biosynthesis. In fact, the biosynthesis of many amino acids was up-regulated by salt stress (Additional file 14: Data S12). Nevertheless, the level of intracellular free amino acids did not increase but instead dropped to certain extents (Additional file 2: Data S1). Probably, the synthesized amino acids are quickly consumed by the algal cells for protein synthesis, as many ribosomal proteins, aminoacyl-tRNA synthetases, translation initiation factors and elongation factors were up-regulated (Additional file 15: Data S13). This is different from the results observed for *C. zofingiensis* under ND conditions [15]. The enhanced protein synthesis is likely a strategy of algal cells for replenishment, as proteins are vulnerable to denaturation caused by salt stress [40, 85, 86]. On the other hand, many chaperones particularly heat-shock proteins were up-regulated (Additional file 16: Data S14), probably preventing and/or reversing the protein denaturation. Notably, the protein catabolism was also stimulated, as indicated by the up-regulation of many proteases and proteasome proteins (Additional file 15: Data S13). This may facilitate the degradation of denatured or less needed proteins and contribute to nitrogen salvage for synthesizing desired proteins to cope with salt stress.

### The salt stress-associated diversion of carbon from starch to storage lipids involves coordinated up-regulation of multiple biological pathways

Starch represents a major carbohydrate reserve in green algae and shares common carbon precursors with the storage lipid TAG. Seemingly, starch serves as a temporary carbon sink and is transformed to TAG for long storage, which has been observed in several algae under ND conditions [15, 89, 100]. It has also been reported that salt stress triggered TAG accumulation at the expense of starch in algae [26, 35, 39, 101], but the underlying mechanism remains rarely touched. We found in *C. zofingiensis* that coordinated up-regulation of multiple biological pathways contributed to the transition of starch to TAG upon salt stress, which are discussed as following.

#### Stimulation of starch catabolism

**Fig. 8:**
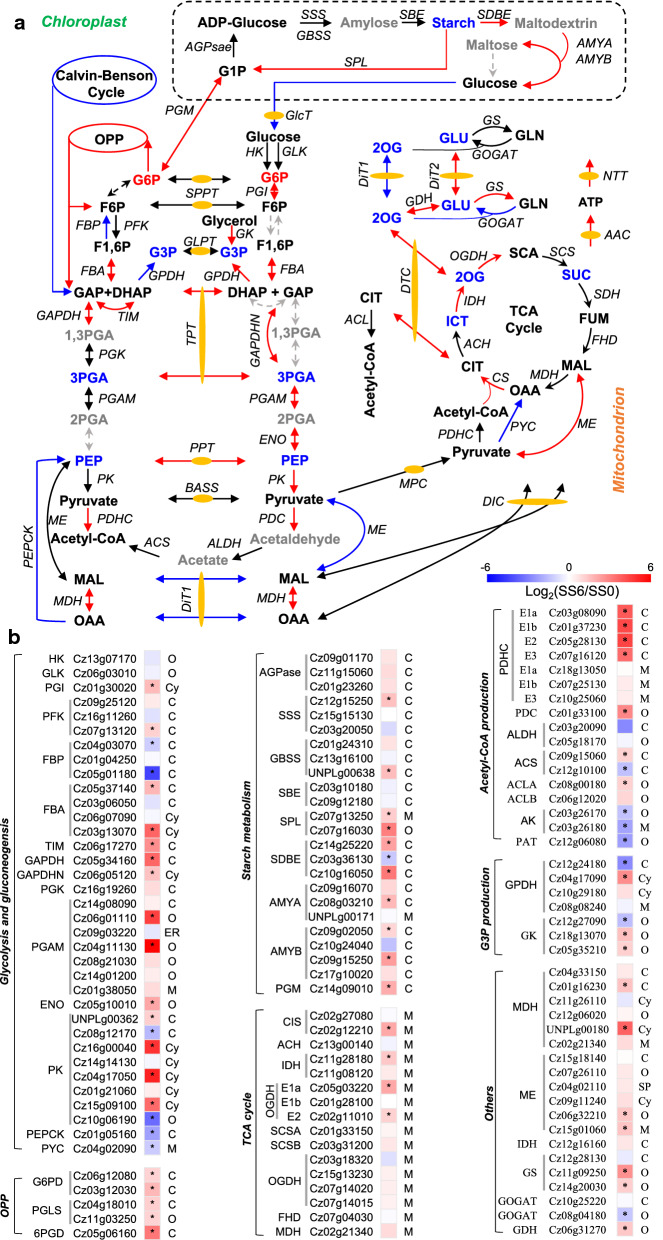
Regulation of central carbon metabolism in response to salinity stress. **a** Transcriptional regulation of central carbon metabolic pathways. Arrows in red, blue, and black indicate transcriptional up-, down-, and non-regulated steps. For proteins encoded by multiple copies of genes, the changes in total transcripts of the isogenes were employed for determining the overall regulation pattern. **b** Heat map showing log2(fold change) values of gene transcripts. Significant difference (at least a twofold change and FDR adjusted p < 0.05) is indicated with an asterisk. Compounds are highlighted with different colors: red, significantly higher; black, not significantly changed; gray not determined; blue, significantly lower under upon salt stress. C chloroplast, *Cy* cytosol, M mitochondrion, *ER* endoplasmic reticulum, *O* other. *AAC* ATP/ADP carrier, *ACH* aconitate hydratase, *ACL* ATP-citrate lyase, *ACS* acetyl-CoA synthetase, *AGPase* ADP-glucose pyrophosphorylase, *ALDH* aldehyde dehydrogenase, *AMYA* alpha-amylase, *AMYB* beta-amylase, *BASS* Bile acid-sodium symporter, *CIS* citrate synthase, *CIT* citrate, *DHAP* dihydroxyacetone phosphate, *DIC* dicarboxylate carrier, *DiT1* 2-oxoglutarate/malate translocator, *DiT2* glutamate/malate translocator, *ENO* enolase, *FBA* fructose-bisphosphate aldolase, *FBP* fructose-1,6-bisphosphatase, *FHD* fumarate hydratase, *F1,6P* fructose-1,6-biphosphate, *F6P* fructose-6-phosphate, *FUM* fumarate, *GAP* glyceraldehyde 3-phosphate, *GAPDHN* glyceraldehyde 3-phosphate dehydrogenase nonphosphorylating, *GAPDH* glyceraldehyde 3-phosphate dehydrogenase, *GBSS* granule bound starch synthase, GK glycerol kinase, *GLK* glucokinase, *GlcT* plastidic glucose transporter, *GLPT* glycerol-3-phosphate transporter, *G1P* glucose-1-phosphate, *G3P* glycerol-3-phosphate, G6P glucose-1-phosphate, *HK* hexokinase, *G6PD* glucose-6-phosphate 1-dehydrogenase, GPDH glycerol-3-phosphate dehydrogenase, *ICT* isocitrate, *MAL* malate, *MDH* malate dehydrogenase, *ME* malic enzyme, *MPC* mitochondrial pyruvate carrier, *NTT* ATP/ADP antiporter, *OAA* oxaloacetate, *2OG* 2-oxoglutarate, OGDH 2-oxoglutarate dehydrogenase, *OPP* oxidative pentose phosphate pathway, *PDHC* pyruvate dehydrogenase complex, *PEP* phosphoenolpyruvate, *PEPCK* phosphoenolpyruvate carboxykinase, *PFK* 6-phosphofructokinase, *1,3PGA* 1,3-bisphosphoglycerate, *2PGA* 2-phosphoglycerate, *3PGA* 3-phosphoglycerate, *PGAM* phosphoglycerate, *PK* pyruvate kinase, *6PGD* 6-phosphogluconate dehydrogenase, *PGI* glucose-6-phosphate isomerase, *PGK* phosphoglycerate kinase, *PGLS* 6-phosphogluconolactonase, *PGM* phosphoglucomutase, *PPT* phosphoenolpyruvate/phosphate translocator, *PYC* pyruvate carboxylase, PDC pyruvate decarboxylase, SBE starch branching enzyme, *SCA* succinyl-CoA, *SPL* starch phosphorylase, *SPPT* sugar phosphate/phosphate translocator, *SCS* succinyl-CoA synthetase, *SDBE* starch debranching enzyme, *SDH* succinate dehydrogenase, *SSS* soluble starch synthase, *SUC* succinate, *TIM* triosephosphate isomerase, *TPT* triose phosphate/phosphate translocator. See Additional file 11: Data S9 for the detailed RNA-seq data

Starch biosynthesis involves a set of enzymes including ADP-glucose pyrophosphorylase (AGPase), starch synthases (SS, both soluble and granule bound) and starch branching enzyme (SBE), of which AGPase is considered as the first committed step [102]. Starch catabolism, on the other hand, has two main pathways leading to the formation of glucose and glucose 1-phosphate (G1P), respectively: the former pathway involves starch debranching enzyme (SDBE) and amylase while the latter is mediated by starch phosphorylase (SPL) (Fig. 8). In *C. zofingiensis*, upon salt stress, genes encoding the enzymes involved in starch biosynthesis had little variation (slight increase) at the transcriptional level; by contrast, the genes in both starch degradation pathways (particularly the SPL-mediated pathway) were up-regulated (Fig. 8; Additional file 11: Data S9). Thus, it is likely that salt stress stimulated starch catabolism while maintaining starch synthesis rate resulting in a decrease in starch content (Fig. 2). This is generally consistent with the phenomenon observed in ND-treated *C. zofingiensis* cells [15], providing carbon building blocks via glycolysis for storage lipids.

#### Stimulation of glycolysis and repression of gluconeogenesis

Similar to *Chlamydomonas* and *Nannochloropsis* [89, 90], *C. zofingiensis* has both chloroplastic and cytosolic glycolysis pathways [15]. Glycolysis is composed of three irreversible steps, namely, glucose phosphorylation catalyzed by hexokinase (HK) or glucokinase (GLK), fructose-6-phosphate (F6P) phosphorylation by 6-phosphofructokinase (PFK), and pyruvate formation by pyruvate kinase (PK). The PK-catalyzed reaction is also the last step of glycolysis and determines the rate of this pathway. In response to salt treatment, a considerable increase (~ 30-fold) in the transcript of *PK* genes was observed (Fig. 8; Additional file 11: Data S9), indicative of the stimulation of glycolysis. Accordingly, phosphoenolpyruvate, the substrate of PK, showed a drop upon salt treatment (Additional file 2: Data S1). Notably, overall, the chloroplastic *PK* genes showed little change while the cytosolic ones were strongly up-regulated (Additional file 11: Data S9), pointing to the dominant contribution of cytosolic glycolysis to pyruvate generation under salt stress conditions. It is worth noting that neither chloroplastic nor cytosolic glycolysis is complete (Fig. 8). Therefore, certain transporters for sugar intermediates are in need to facilitate the completion of glycolysis, such as triose phosphate/phosphate translocator (TPT) and phosphoenolpyruvate/phosphate translocator (PPT), which were up-regulated by salt (Additional file 12: Data S10). As an opposite pathway to glycolysis, gluconeogenesis has four irreversible steps, catalyzed by pyruvate carboxylase (PYC), phosphoenolpyruvate carboxykinase (PEPCK), fructose-1,6-bisphosphatase (PBP), and G6P phosphatase. The genes encoding the first three enzymes were identified in *C. zofingiensis* and all showed a considerable decrease at the transcriptional level upon salt treatment (Fig. 8; Additional file 11: Data S9), suggesting the repression of gluconeogenesis. Thus, by stimulating glycolysis and repressing gluconeogenesis, salt stress drove carbon flux from starch degradation products to pyruvate for the downstream production of acetyl-CoA, the precursor of de novo fatty acid synthesis.

#### Enhanced biosynthesis of acetyl-CoA and G3P

Acetyl-CoA has multiple sources in different cell compartments (e.g., chloroplast, cytosol and mitochondria): (1) from pyruvate catalyzed by pyruvate dehydrogenase complex (PDHC); (2) from acetate by acetyl-CoA synthetase (ACS) or by acetate kinase (AK) and phosphate acetyltransferase (PAT); (3) from citrate via ATP-citrate lyase (ACL), etc. [103]. While mitochondrial acetyl-CoA feeds into the TCA cycle, chloroplastic acetyl-CoA is used directly for de novo fatty acid synthesis [103]. In *C. zofingiensis*, the production of chloroplastic acetyl-CoA relies on chloroplast-targeted PDHC and ACS [15]. Upon salt stress, the subunits of chloroplastic PDHC were up-regulated considerably (over 10-fold increase) in a well-coordinated manner; although ACS overall had little change, pyruvate decarboxylase (PDC) that provides acetate for ACS were up-regulated (Fig. 8 and Additional file 13: Data S11), pointing to the enhanced synthesis of chloroplastic acetyl-CoA. Probably, the synthesized acetyl-CoA is utilized quickly by the de novo fatty acid synthesis, which was stimulated by salt stress (Fig. 4), leading to no observed accumulation of acetyl-CoA (Additional file 2: Data S1).

G3P, the backbone of glycerolipids, can be from dihydroxyacetone phosphate (DHAP) mediated by G3P dehydrogenase (GPDH). *C. zofingiensis* possesses four GPDH-encoded genes; the chloroplastic (Cz12g24180) and mitochondrial (Cz08g08240) ones were down-regulated, while the two cytosolic ones (Cz04g17090 and Cz10g29180) were up-regulated (fivefold increase for Cz04g17090) by salt stress (Fig. 8 and Additional file 13: Data S11). This is generally consistent with the results under ND conditions that Cz04g17090 was up-regulated [15], indicative of its critical role in G3P production for glycerolipid assembly. Interestingly, in *C. reinhardtii*, the chloroplastic *GPDH* genes rather than the cytosolic ones were up-regulated by ND or salt stress, and played a role in TAG accumulation [98, 104]. This may partially explain that under TAG induction conditions, *C. zofingiensis* activates transcriptional up-regulation of the extrachloroplastic GPAT (Fig. 6; [15]), while *C. reinhardtii* activates transcriptional up-regulation of the chloroplastic one [89]. G3P can also be derived from glycerol mediated by glycerol kinase (GK). Seemingly, the contribution of GK to G3P provision is marginal under salt stress, as *C. zofingiensis* showed little change in the overall transcript level of *GK* genes (Additional file 13: Data S11). Despite the stimulation of G3P synthesis, its level saw a drop upon salt stress (Additional file 2: Data S1), likely because it is consumed for TAG assembly and glycerol production (mediated by glycerol-3-phosphatase), which were both up-regulated (Fig. 6; Additional file 8: Data S6 and Additional file 13: S11).

#### Stimulated generation of reductant and energy

De novo synthesis and desaturation of fatty acids require input of substantial amounts of reductant (e.g., NADPH) and energy (e.g., ATP). The considerable up-regulation of fatty acid synthetic pathways (Fig. 4) and increase of fatty acids (Fig. 2) suggested the need of stimulated provision of NADPH and ATP under salt stress conditions. Probably, multiple sources are stimulated to meet NADPH need in *C. zofingiensis*. Firstly, ferredoxin NADP reductase (FNR), catalyzing the NADPH-producing step in photosynthesis, was up-regulated (Additional file 13: Data S11). This is interesting and different from the results under ND conditions where *FNR* gene was down-regulated in algae [15, 56, 90]. Secondly, the enzymes responsible for the two NADPH-producing steps in the oxidative pentose phosphate (OPP) pathway, glucose-6-phosphate dehydrogenase (G6PDH) and 6-phosphogluconate dehydrogenase (6PGD), were all up-regulated at the transcriptional level (Additional file 13: Data S11), in agreement with the results under ND conditions [15]. Besides, some NADP^+^-dependent enzymes may contribute to the reductant generation, such as malic enzyme (ME; Cz06g32210), malate dehydrogenase (MDH; Cz01g16230) and glyceraldehyde 3-phosphate dehydrogenase (GAPDH; Cz06g05120), which were up-regulated mildly (Additional file 13: Data S11). Considering the abundance and up-regulation extent of these NADPH-producing enzymes at the transcriptional level (Additional file 13: Data S11), the OPP pathway may play a major role in NADPH supply in *C. zofingiensis* under salt stress conditions. As for ATP, it is likely contributed by substrate-level phosphorylation in glycolysis and the TCA cycle under salt stress conditions: (1) up-regulation of PKs that catalyze the last step of glycolysis for ATP generation; (2) up-regulation of isocitrate dehydrogenase (IDH) that produces ATP, and of 2-oxoglutarate dehydrogenase (OGDH) that produces NADH for ATP generation via oxidative phosphorylation (Fig. 8; Additional file 13: Data S11). Accordingly, the metabolites involved in the TCA cycle (e.g., isocitrate, 2-oxoglutarate and succinate) were decreased (Additional file 2: Data S1), which is generally consistent with the ND-induced results [15, 105]. Meanwhile, ATP transporters such as mitochondrial ATP/ADP carrier (AAC) that transports ATP from mitochondria to cytosol and chloroplastic ATP/ADP antiporter that transports ATP from cytosol to chloroplast [106], were up-regulated to meet the enhanced consumption of ATP for fatty acid synthesis in the chloroplast (Fig. 8; Additional file 12: Data S10).

### *C. zofingiensis* synthesizes TAG and astaxanthin in a coordinated way

TAG and astaxanthin are secondary metabolites and generally accumulate under unfavorable growth conditions in algae. The simultaneous accumulation of TAG and astaxanthin has been observed in *H. pluvialis* [8, 13]. Similarly, *C. zofingiensis* synthesizes TAG and astaxanthin simultaneously under ND, high light, or the combination of the two stress conditions [11]. This was also observed in response to salt stress, with a high coefficient of 0.998 (Additional file 1: Figure S2), pointing to the coordinated synthesis of TAG and astaxanthin regardless of stress conditions in *C. zofingiensis*. Nevertheless, it is worth noting that TAG levels are much higher (ca. 100-fold) than astaxanthin levels (Additional file 1: Figure S2), suggesting the predominant carbon flux towards TAG compared to astaxanthin. The presence of crosstalk has been proposed between TAG and astaxanthin biosynthesis [8, 11]. First, algal TAG and astaxanthin may compete with each other for carbon precursors. It has been reported that impairing TAG accumulation via de novo fatty acid synthesis inhibitor led to enhanced astaxanthin level in *C. zofingiensis* [11]. Second, astaxanthin, predominantly esterified with fatty acids in algae, is stored in LDs that consist of a TAG-filled hydrophobic core [76]. Probably, a basal level of TAG is required to build LDs for astaxanthin storage [8, 13]. The astaxanthin-stored LDs, peripherally scattered within *C. zofingiensis* cells [16], likely function as a ‘sunscreen’ to alleviate photodamage. TAG and astaxanthin in LDs may also serve as the so-called compatible solutes to help algal cells cope with the osmotic stress caused by salt. The synthesis of TAG and astaxanthin in *C. zofingiensis* might be subjected to coordinated regulation by such regulators as transcription factors (TFs). In response to salt stress, of the 180 putative TFs, 19 were up-regulated and 48 were down-regulated (Additional file 17: Data S15). MYB (Cz10g24240) and bHLH (UNPLg00160), which might regulate both TAG and astaxanthin biosynthesis based on co-expression analysis [15], were up-regulated by salt stress (Additional file 17: Data S15), consistent with the expression pattern of key genes involved in TAG and astaxanthin synthesis, e.g., *GPAT2*, *LPAAT1*, *PAP3*, *DGTT5*, *MLDP*, *LCYb*, *BKT1* (Additional file 8: Data S6 and Additional file 9: Data S7). In this context, these TFs are potential engineering targets for improving both TAG and astaxanthin in *C. zofingiensis* and worthy of further investigation.

### *C. zofingiensis* has distinctions in oleaginousness and carotenogenesis between salt stress and nitrogen deprivation conditions

**Fig. 9:**
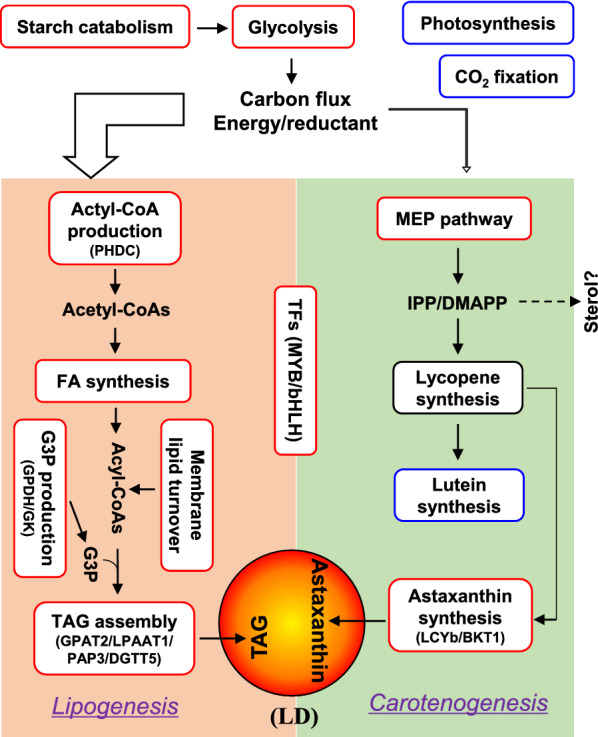
A mechanistic model for the salt stress-induced lipogenesis and carotenogenesis in *C. zofingiensis*. Boxes in red, blue, and black indicate up-, down-, and non-regulated pathways, respectively. The thickness of the right angle arrow designates the flux of carbon, energy and reductant allocated for lipogenesis and carotenogenesis (not on scale)

Both salt stress and ND conditions can activate certain biological pathways (Table 1) and trigger the accumulation of TAG and astaxanthin in *C. zofingiensis* (Fig. 2; [9, 11, 14]). Yet there are distinctions in oleaginousness for TAG synthesis between the two conditions (Table 1): (1) under salt stress conditions, the chloroplastic PDHC likely plays a major role in the production of acetyl-CoA in chloroplast for de novo fatty acid synthesis, while under ND conditions, ACS may also contribute; (2) as for the fatty acid desaturation, seemingly ND rather than salt stress activates up-regulation of all FADs; (3) of the enzymes involved in TAG assembly, only the chloroplastic LPAAT is up-regulated by salt stress while both chloroplastic and extrachloroplastic ones are up-regulated by ND, the extrachloroplastic PAP is up-regulated by salt stress while the chloroplastic one is up-regulated by ND, and only the type II DGAT (DGTT5) is up-regulated by salt stress while both type I and type II DGATs (DGAT1A, DGAT1B, and DGTT5 through DGTT8) are up-regulated by ND. In this context, salt stress seemingly stimulates less than ND in ‘pushing’ (pushes carbon flux to precursors for lipid metabolism) and ‘pulling’ (pulls fatty acids to glycerol backbone for TAG assembly), thus leading to a lower TAG level (Fig. 1; [11]). *C. zofingiensis* also shows variations in astaxanthin biosynthesis between salt stress and ND conditions. The conversion of β-carotene to astaxanthin involves two types of enzymes, BKT and CHYb [16]. Only BKT1 is activated upon salt stress while both BKTs (BKT1 and BKT2), CHYb and AAT are activated by ND (Table 1), which may explain why *C. zofingiensis* accumulates more astaxanthin under ND conditions (Fig. 1; [11]). Furthermore, the comparison reveals the critical genes that contribute to TAG and astaxanthin build-up regardless of stress conditions, such as *GPAT2*, *LPAAT1*, *DGTT5*, and *BKT1*, which are potential candidates for manipulation to improve the traits of *C. zofingiensis* once an advanced genetic toolbox is established.

**Table 1:** Comparison of the up-regulated genes involved in selected pathways of *C. zofingiensis* between salt stress and ND conditions

Pathways	Salt stress	ND
Acetyl-CoA production	PDHC (C), PDC, ACL	PDHC (C), PDC, ACS, ACL, AK
De novo fatty acid synthesis	ACCase complex, ACP, MCT, KAR, HAD, ENR, KAS I, KAS II, KAS III	ACCase complex, ACP, MCT, KAR, HAD, ENR, KAS I, KAS II, KAS III
Fatty acid desaturation	SAD, FAD2, FAD5, FAD6, FAD7	SAD, FAD2, FAD4, FAD5, FAD6, FAD7, ∆4/∆6FAD
Membrane lipid turnover	PGD1	PGD1
G3P and acyl-CoA production	GK, GPDH (cyto), FAT, LCAS (C)	GK, GPDH (cyto), FAT, LCAS (C)
TAG assembly	GPAT2 (ER), LPAAT1 (C), PAP3 (ER), DGTT5, PDAT	GPAT2 (ER), LPAAT1, 2 (C, ER), PAP1 (C), DGAT1, DGTT (1, 5-8)
IPP/DMAPP production	MCS, HDS, HDR	MCS, AACT, HCS
Carotenoid synthesis	LCYb, BKT1	LCYb, BKT1, BKT2, CHYb, AAT
NADPH production	FNR, G6PD, 6PGD, MDH	G6PD, 6PGD, ME
Energy production	PK (cyto; ATP), GAPDH (NADH), IDH (NADH), OGDH (NADH)	PK (C, cyto; ATP), GAPDH (NADH), SCS (ATP), IDH (NADH), OGDH (NADH)

The gene expression data for *C. zofingiensis* under ND conditions were retrieved from Liu et al. [15]. Cut-off for up-regulation: fold change > 2 and adjusted p < 0.05. Subcellular localization information: *cyto* cytosol, *C* chloroplast, *ER* endoplasmic reticulum, *M* mitochondria. *AAT* long-chain-alcohol *O*-fatty-acyltransferase, *ACCase* acetyl-CoA carboxylase, *ACP* acyl carrier protein, *ACS* acetyl-CoA synthetase, *AK* acetate kinase, *ATL* ATP-citrate lyase, *DGAT* diacylglycerol *O*-acyltransferase type I, DGTT diacylglycerol *O*-acyltransferase type II, *ENR* enoyl-ACP reductase, *FAD* fatty acid desaturase, *FAT* acyl-ACP thioesterase, *FNR* ferredoxin NADP reductase, *GK* glycerol kinase, *GAPDH* glyceraldehyde 3-phosphate dehydrogenase, *GPAT* glycerol-3-phosphate acyltransferase, *G6PD* glucose-6-phosphate 1-dehydrogenase, *6PGD* 6-phosphogluconate dehydrogenase, *GPDH* glycerol-3-phosphate dehydrogenase, *HAD* 3-ketoacyl-ACP dehydratase, *IDH* isocitrate dehydrogenase, *KAR* 3-ketoacyl-ACP reductase, *KAS* 3-ketoacyl-ACP synthase, *LCAS* long-chain acyl-CoA synthetase, *LPAAT* 1-acyl-sn-glycerol-3-phosphate acyltransferase, *MCT* malonyl-CoA:acyl carrier protein transacylase, *ME* malic enzyme, *MDH* malate dehydrogenase, *OGDH* 2-oxoglutarate dehydrogenase, *PAP* phosphatidate phosphatase, *PDAT* phospholipid:diacylglycerol acyltransferase, *PDC* pyruvate decarboxylase, *PDHC* pyruvate dehydrogenase complex, *PGD1* plastid galactoglycerolipid degradation 1, *PK* pyruvate kinase, *SAD* stearoyl-ACP desaturase, *SCS* succinyl-CoA synthetase

## Conclusions

Among the tested salt concentrations, 0.2 M was demonstrated to maximize both TAG and astaxanthin contents and their productivities in *C. zofingiensis*. Multi-omics analysis revealed a global response of *C. zofingiensis* to salt stress, including attenuated photosynthesis and CO_2_ fixation, accelerated protein turnover, remodeled central carbon metabolism, oleaginousness and carotenogenesis, which provided a solid basis for better understanding TAG and astaxanthin synthesis in the alga (Fig. 9). The coordinated up-regulation of multiple pathways, such as carbon shunt from starch, acetyl-CoA production, fatty acid synthesis, membrane lipid turnover, G3P production, TAG assembly and LD formation, provided a strategic combination of ‘pushing’, ‘pulling’ and ‘protection’ to realize TAG accumulation. Astaxanthin, on the other hand, was induced to accumulate mainly by stimulating ‘pulling’ and ‘protection’ (up-regulation of *LCYb*, *BKT1* and *AAT*) that diverted carotenoid flux from lutein to astaxanthin, which was esterified with fatty acids and packed into LDs for storage. TAG and astaxanthin accumulated in a coordinated manner, probably regulated by such TFs as MYB and bHLH. Comparative analysis between salt stress and ND conditions disclosed distinctions in addition to the common features with respect to oleaginousness and carotenogenesis of *C. zofingiensis*. Furthermore, critical enzymes and regulators for TAG and astaxanthin biosynthesis were identified. These results together (1) demonstrate the beneficial effect of salt on TAG and astaxanthin synthesis in *C. zofingiensis* and point to the potential of using diluted seawater for production uses; (2) shed light on the mechanisms of oleaginousness (for TAG boost) and carotenogenesis (for astaxanthin accumulation), and (3) provide candidate gene targets for future trait improvements via rational genetic engineering. It should be noted that the significant transcript change of a gene does not always guarantee its critical function and additional evidences (protein level, in vitro, in vivo, etc.) are needed.

## Methods

### Algal strain and growth conditions

*Chromochloris zofingiensis* (ATCC 30,412) was purchased from the American Type Culture Collection (ATCC, Rockville, MD, USA). To recover algal activity, the cells from the maintaining alga was inoculated into flasks grown aerobically at 25 °C for 6 days with orbital shaking (150 rpm) and continuous illumination (30 µE m^−2^ s^−1^). The cells were then inoculated at 10% (v/v) into a 200-mL column (3-cm diameter), and grown to exponential phase under constant illumination of 70 µE m^−2^ s^−1^ and aeration of 1.5% CO_2_ enriched air. The algal cells in exponential phase were harvested by centrifugation, resuspended in fresh medium supplemented with different sodium chloride concentrations (0, 0.1, 0.2, 0.4 and 0.6 M), and allowed to grow in columns under the same conditions mentioned above.

### Determination of physiological and biochemical changes

Cell number was determined under a light microscope by using a hemocytometer. Dry weight was determined by weighing pre-dried Whatman GF/C filter papers (1.2 μm pore size). *F*v/*F*m, the potential quantum efficiency of PSII indicating the photosynthetic performance, was measured in a pulse amplitude-modulated (PAM) fluorometry (Walz, Germany) as previously stated [15].

Cell samples were harvested by centrifugation and lyophilized on a freeze-drier (Labconco, MO, USA) for subsequent biochemical analysis. Protein was extracted and determined as described by Liu et al. [15]. Starch was determined using the Starch Assay Kit (Sigma-Aldrich, MO, USA) according to the manual’s instructions.

Lipids were extracted with chloroform–methanol (2:1) and the total lipids were determined gravimetrically [15]. Neutral lipids and polar lipids were separated on a Silica gel 60 TLC plate (EMD Chemicals, Merck, Germany) with different mobile phases: the former used a mixture of hexane/tert-butylmethyl ether (TBME)/acetic acid (80/20/2, by vol), while the latter used a mixture of chloroform/methanol/acetic acid/water (25/4/0.7/0.3, by vol) [53]. Total lipids or individual lipids recovered from TLC plates were transesterified with sulfuric acid in methanol [11]. Fatty acid methyl esters (FAMEs) were analyzed by using an Agilent 7890 capillary gas chromatograph equipped with a 5975 C mass spectrometry detector and a HP-88 capillary column (60 m × 0.25 mm) (Agilent Technologies, CA, USA) as detailed by Mao et al. [63]. Individual FAMEs were quantified with authentic standards in the presence of the internal standard heptadecanoic acid (Sigma-Aldrich). The content of TAG and polar lipids was expressed as the content of their corresponding fatty acids.

Carotenoid extraction and determination followed the procedures described in our previous study [11]. Briefly, the lyophilized cell samples were homogenized vigorously in the presence of liquid nitrogen and extracted with acetone for three times under dim light. The carotenoid extracts were then separated on a high performance liquid chromatography system, which is composed of a Waters 2695 separation module, a Waters 2996 photodiode array detector and a Waters Spherisorb 5 µm ODS2 4.6 × 50 mm analytical column (Waters, MA, USA). For the ideal separation of lutein and zeaxanthin, a Waters YMC Carotenoid C30 column (5 μm, 4.6 × 250 mm) was used. Carotenoids were identified and quantified by comparing with authentic standards regarding the retention time, absorption spectra and peak area.

Metabolites were extracted with cold 80% methanol by using a mini-beadbeater (Biospec Products, OK, USA) and analyzed by the Metabolomics Facility at Technology Center for Protein Sciences, Tsinghua University, using a TSQ Quantiva Triple Quadrupole liquid chromatography–mass spectrometry (Thermo Scientific) equipped with a 100 × 3 mm Synergi™ Hydro-RP 100A column (Phenomenex, CA, USA) according to our previously described procedures [15]. Relative quantification was employed between biological conditions (0 and 12 h of salt stress) wherein the data from 0 h served as the reference control. Significant difference was achieved when the relative value showed at least a 1.5-fold change with a *p-*value less than 0.05 (Student’s *t*-test).

### RNA-seq and analysis of differentially expressed genes

RNA was extracted from cell samples under the two conditions (0 and 6 h of salt stress) using the plant RNA extraction kit (TaKaRa, Japan) according to the manufacturer’s instructions. Contaminated DNA was removed by treating with RNase-free DNase I (TaKaRa). The RNA quality and concentration were examined using an Agilent 2100 Bioanalyzer (Agilent Technologies) and a NanoDrop 2000C (Thermo Scientific, DE, USA). Around 10 mg total RNA was used for mRNA purification with Sera-mag Magnetic Oligo(dT) Beads (Thermo Scientific). The transcriptome libraries were prepared using the NEBNext mRNA Library Prep Reagent Set (New England Biolabs, MA, USA) according to the manual’s instructions and sequenced on a BGISEQ-500 platform (BGI, China). The RNA-seq raw data were deposited in the Gene Expression Omnibus under accession number GSE125419.

Clean reads (obtained by filtering the low-quality reads, reads with adaptors and reads with unknown bases) were aligned to *C. zofingiensis* genome [18] by using TopHat (version 2.0.4), allowed no more than two segment mismatches. Gene expression was measured as the numbers of aligned reads to annotated genes by Cufflinks (version 2.0.4) and normalized to the number of Fragments Per Kilobase Million (FPKM). Differentially expressed genes (DEGs) in response to salt stress were defined as follows: the FPKM value of at least one condition was no less than 1 and gene expression showed at least a twofold change with the false discovery rate (FDR) adjusted *p*-value less than 0.05. The DEGs were manually grouped into 12 categories to analyze the possible functional enrichment as previously described [90], in addition to KEGG pathway enrichment analysis.

### Quantitative real-time PCR for the validation of RNA-seq data

Total RNA (1 μg) extracted from cell samples under different conditions (0, 6, 12, 24 and 48 h of salt stress) was reversely transcribed to cDNA by using the PrimeScript™ RT Master Mix (TaKaRa, Japan) according to the manufacturer’s instructions. Quantitative real-time PCR (qPCR) was performed using a 7500 Fast Real-Time PCR System (Applied Biosystems, MA, USA) with SYBR^®^ Premix Ex Taq™ II (TaKaRa, Japan). A total of 24 genes regarding lipid metabolism, astaxanthin biosynthesis and TFs were analyzed and their qPCR primers are listed in Additional file 3: Table S2. The housekeeping gene β-actin was used as the internal control, and the relative gene expression level was calculated based on the 2^−ΔΔ*C*t^ method [107].

## Supplementary information

**Additional file 1: Figure S1.** Time course of percentage of SFA, UFA, and MUFA in response to 0.2 M NaCl. SFA, saturated fatty acids; UFA, unsaturated fatty acids; MUFA, monounsaturated fatty acids. **Figure S2.** Correlation analysis between primary and secondary carotenoids, esterified astaxanthin and TAG, and esterified astaxanthin and oleic acid. The data were from the time-resolved experiment under 0.2 M salt concentration. **Figure S3.** Time course distribution of free astaxanthin, mono-ester, and di-ester in response to 0.2 M NaCl. **Figure S4.** Consistency between RNA-seq–based and qPCR–based transcript quantification. A total of 24 genes were chosen for qPCR validation. The genes and primer sequences for qPCR are listed in Additional file 3: Table S2. **Figure S5.** The relative abundance of C16:0 and C18:1 in *sn*-2 position of TAG from *C. zofingiensis* under ND and SS conditions. ND, nitrogen deprivation; SS, 0.2 M salt. Asterisk indicates the significant difference (*t*-test, p < 0.05) between ND and SS. **Figure S6.** Time course of chlorophyll content in *C. zofingiensis* in the presence of 0.2 M salt.

**Additional file 2: Data S1.** Change of metabolites in *C. zofingiensis* in response to salt stress.

**Additional file 3: Table S1.** Reads quality and mapping ratio of the transcriptomes. **Table S2.** The primer sequences of selected genes used in qPCR experiments.

**Additional file 4: Data S2.** List of FPKM values of all genes.

**Additional file 5: Data S3.** List of 6473 DEGs.

**Additional file 6: Data S4.** KEGG pathway functional enrichment of DEGs.

**Additional file 7: Data S5.** Manual curation of DEGs.

**Additional file 8: Data S6.** RNA-seq data for genes involved in lipid metabolism.

**Additional file 9: Data S7.** RNA-seq data for genes involved in carotenogenesis.

**Additional file 10: Data S8.** RNA-seq data for genes involved photosynthesis.

**Additional file 11: Data S9.** RNA-seq data for genes involved in CO_2_ fixation and central carbon metabolism.

**Additional file 12: Data S10.** RNA-seq data for genes involved in ROS scavenging and transport of certain metabolites.

**Additional file 13: Data S11.** RNA-seq data for genes involved in producing acyl-CoAs, G3P, reductant and energy molecules.

**Additional file 14: Data S12.** RNA-seq data for genes involved in amino acid metabolism.

**Additional file 15: Data S13.** RNA-seq data for genes involved in protein metabolism.

**Additional file 16: Data S14.** RNA-seq data for genes encoding putative chaperones.

**Additional file 17: Data S15.** RNA-seq data for genes encoding putative transcription factors.

## Data Availability

All data generated or analyzed during this study are included in this published article and its supplementary information files.

## Abbreviations

AACT: Acetoacetyl-CoA thiolase
AAH: Allantoin amidohydrolase
AAT: Long-chain-alcohol O-fatty-acyltransferase
AMI: Formamidase
AMT: Ammonium transporter
AMX: Amine oxidase
ATDI: Allantoate deiminase
BKT: Beta-carotenoid ketolase
BPGA: 1,3-Bisphosphoglycerate
BUP: β-Ureidopropionase
CDP-ME: 4-Diphosphocytidyl-2-C-methylerythritol
CDP-MEP: 4-Diphosphocytidyl-2-C-methyl-d-erythritol 2-phosphate
CHYb: Beta-carotenoid hydroxylase
CMK: 4-Diphosphocytidyl-2-C-methyl-d-erythritol kinase
CMS: 2-C-Methyl-d-erythritol 4-phosphate cytidylyltransferase
CRTISO: Carotenoid isomerase
CYP97A: Cytochrome P450 beta hydroxylase
CYP97C: Cytochrome P450 epsilon hydroxylase
DEG: Differentially expressed gene
DHAP: Dihydroxyacetone phosphate
DHDH: Dihydrouracil dehydrogenase
DHP: Dihydropyrimidinase
DMAPP: Dimethylallyl pyrophosphate
DUR1: Urea carboxylase
DUR2: Allophanate hydrolase
DUR3: Urea active transporter
DXR: 1-Deoxy-d-xylulose 5-phosphate reductoisomerase
DXP: 1-Deoxy-d-xylulose 5-phosphate
DXS: 1-Deoxy-d-xylulose 5-phosphate synthase
E4P: Erythrose 4-phosphate
FBA: Fructose-bisphosphate aldolase
FBPase: Fructose-1,6-bisphosphatase
FBP: Fructose 1,6-bisphosphate
FDR: False discovery rate
F6P: Fructose 6-phosphate
Fd: Ferredoxin
FNR: Ferredoxin-NADP (+) reductase
FPP: Farnesyl diphosphate
FPPS: Farnesyl diphosphate synthase
GAP: Glyceraldehyde 3-phosphate
GAPDH: Glyceraldehyde 3-phosphate dehydrogenase
GDA: Guanine deaminase
GDH: Glutamate dehydrogenase
Glu: Glutamate
Gln: Glutamine
GOGAT: Glutamate synthase
GGPP: Geranylgeranyl diphosphate
GGPPS: Geranylgeranyl diphosphate synthase
GPP: Geranyl diphosphate
GPPS: Geranyl diphosphate synthase
GS: Glutamine synthetase
HCR: HMG-CoA reductase
HCS: Hydroxymethylglutaryl-CoA synthase
HDR: 4-Hydroxy-3-methylbut-2-en-1-yl diphosphate reductase
HDS: 4-Hydroxy-3-methylbut-2-en-1-yl diphosphate synthase
HGM-CoA: 3-Hydroxy-3-methylglutaryl-CoA
HMB-PP: (E)-4-Hydroxy-3-methyl-but-2-enyl pyrophosphate
hν: Photon energy
IPP: Isopentenyl pyrophosphate
IPPI: Isopentenyl-diphosphate Delta-isomerase
LCYb: Lycopene beta cyclase
LCYe: Lycopene epsilon cyclase
LHC: Light harvesting complex
MCS: 2-C-Methyl-d-erythritol 2,4-cyclodiphosphate synthase
MDH: Malate dehydrogenase
ME: Malic enzyme
MEcPP: 2-C-Methyl-d-erythritol 2,4-cyclodiphosphate
MEP: 2-C-Methylerythritol 4-phosphate
MK: Mevalonate-5-kinase
MPK: Phosphomevalonate kinase
MPPD: Mevalonate-5-pyrophosphate decarboxylase
NAR1: Nitrate transporter: NAR1 family
NAR2: Nitrate transporter: NAR2 family
ND: Nitrogen deprivation
NIR: Nitrite reductase
NR: Nitrate reductase
NRT1: Nitrate transporter: NRT1 family
NRT2: Nitrate transporter: NRT1 family
NPQ: Non-chemical quenching
NXS: Neoxanthin synthase
OAA: Oxaloacetate
2OG: 2-Oxoglutarate
PC: Plastocyanin
PDS: Phytoene desaturase
PEP: Phosphoenolpyruvate
PEPC: Phosphoenolpyruvate carboxylase
3PGA: 3-Phosphoglycerate
PGK: Phosphoglycerate kinase
PPDK: Pyruvate phosphate dikinase
PQ: Plastoquinone
PRK: Phosphoribulokinase
PS: Photosystem
PSY: Phytoene synthase
RBCS: Ribulose-1,5-bisphosphate carboxylase/oxygenase small subunit
ROS: Reactive oxygen species
R5P: Ribose 5-phosphate
RPH: Ammonium transporter: Rh family
RPI: Ribose 5-phosphate isomerase
RPE: Ribulose-phosphate 3-epimerase
RuBP: Ribulose 1,5-bisphosphate
Ru5P: Ribulose 5-phosphate
SBP: Sedoheptulose 1,7-bisphosphate
SBPase: Sedoheptulose-1,7-bisphosphatase
S7P: Sedoheptulose 7-phosphate
TAG: Triacylglycerol
TIM: Triosephosphate isomerase
TRK: Transketolase
UAH: Ureidoglycolate amidohydrolase
UIAH: Ureidoglycine aminohydrolase
UOX: Urate oxidase
VDE: Violaxanthin de-epoxidase
XDH: Xanthine dehydrogenase
Xu5P: Xylulose 5-phosphate
ZDS: Zeta-carotene desaturase
ZEP: Zeaxanthin epoxidase
ZISO: Zeta-carotene isomerase
