# Correction: Novel insights into salinity-induced lipogenesis and carotenogenesis in the oleaginous astaxanthin-producing alga *Chromochloris zofngiensis*: a multi-omics study

**DOI:** 10.1186/s13068-023-02282-7

**Published:** 2023-03-07

**Authors:** Xuemei Mao, Yu Zhang, Xiaofei Wang, Jin Liu

**Affiliations:** Laboratory for Algae Biotechnology and Innovation, College of Engineering, Peking University, Beijing, 100871 China

**Correction** **:** **Biotechnol Biofuels (2020) 13:73** 10.1186/s13068-020-01714-y

Following publication of the original article [1], the authors noticed an error in the grant number under Funding section. The correct funding number should be 2018YFA0902500 instead of 2018YFA090250. This has been corrected in this correction.

Funding

This work is partially supported by grants from National Key R&D Program of China (2018YFA0902500), National Youth Thousand Talents Program of China and Peking University CCUS project supported by BHP Billiton.

The original article [1] has been corrected.
