# Supplementary material for: Novel insights into salinity-induced lipogenesis and carotenogenesis in the oleaginous astaxanthin-producing alga Chromochloris zofingiensis: a multi-omics study
Source: Biotechnol Biofuels. 2020 Apr 16;13:73. doi: 10.1186/s13068-020-01714-y (PMC7161124; doi:10.1186/s13068-020-01714-y)
Supplement: Supplementary file 1 — Additional file 1: Figure S1. Time course of percentage of SFA, UFA, and MUFA in response to 0.2 M NaCl. SFA, saturated fatty acids; UFA, unsaturated fatty acids; MUFA, monounsaturated fatty acids. Figure S2. Correlation analysis between primary and secondary carotenoids, esterified astaxanthin and TAG, and esterified astaxanthin and oleic acid. The data were from the time-resolved experiment under 0.2 M salt concentration. Figure S3. Time course distribution of free astaxanthin, mono-ester, and di-ester in response to 0.2 M NaCl. Figure S4. Consistency between RNA-seq–based and qPCR–based transcript quantification. A total of 24 genes were chosen for qPCR validation. The genes and primer sequences for qPCR are listed in Additional file 3: Table S2. Figure S5. The relative abundance of C16:0 and C18:1 in sn-2 position of TAG from C. zofingiensis under ND and SS conditions. ND, nitrogen deprivation; SS, 0.2 M salt. Asterisk indicates the significant difference (t-test, p < 0.05) between ND and SS. Figure S6. Time course of chlorophyll content in C. zofingiensis in the presence of 0.2 M salt. [file 13068_2020_1714_MOESM1_ESM.pdf]

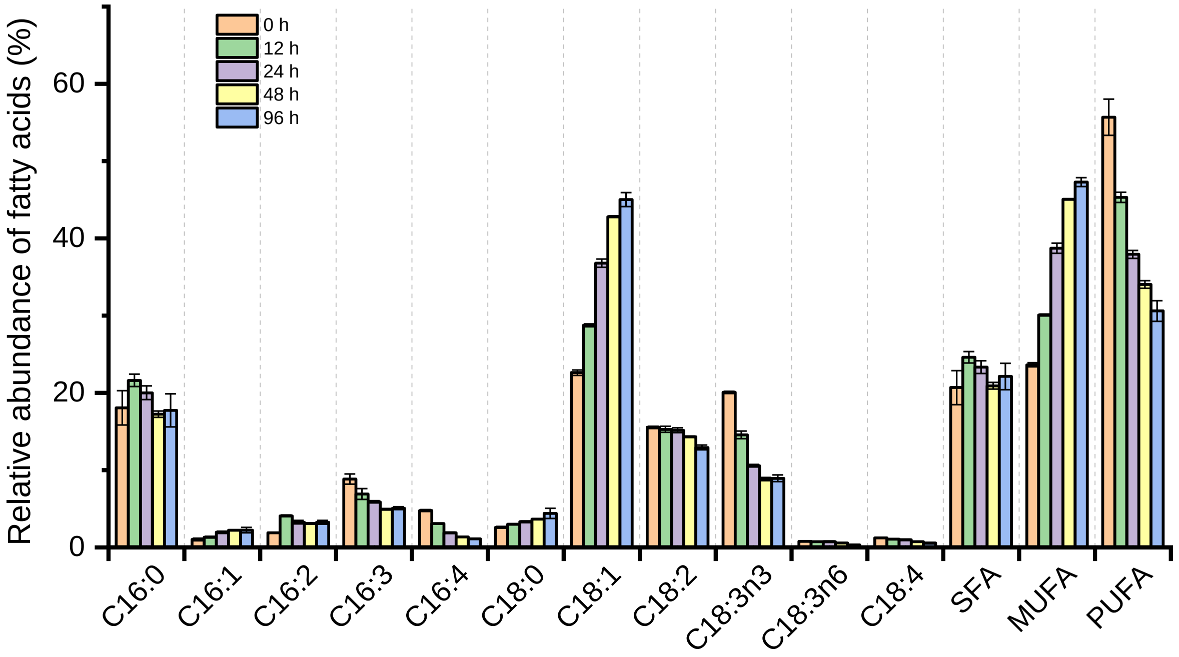

Figure S1. Time course of relative abundance of fatty acids in response to 0.2 M NaCl. SFA, saturated fatty acids; MUFA, monounsaturated fatty acids; PUFA, polyunsaturated fatty acids.

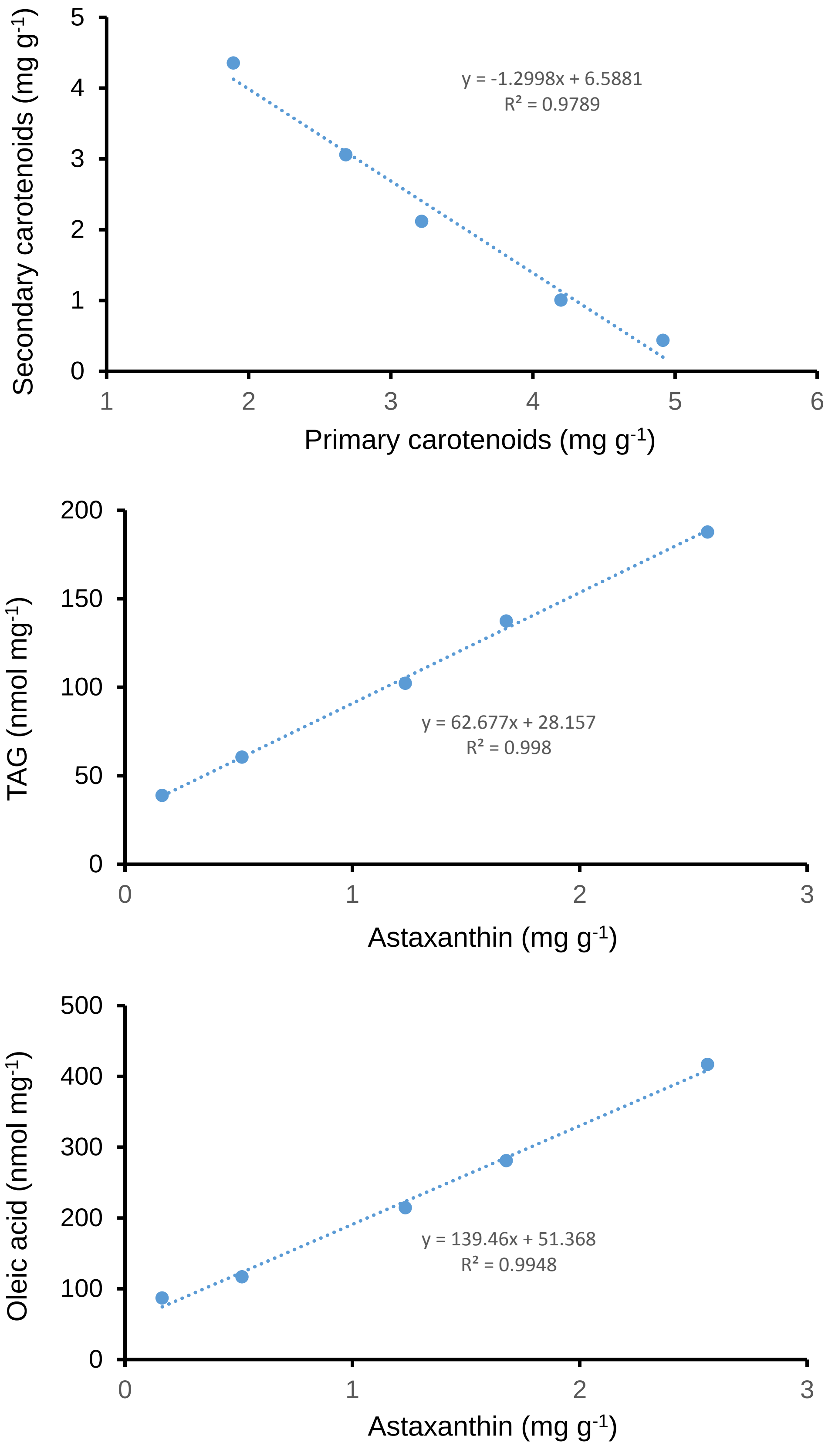

Figure S2. Correlation analysis between primary and secondary carotenoids, esterified astaxanthin and TAG, and esterified astaxanthin and oleic acid. The data were from the time-resolved experiment under 0.2 M salt concentration.

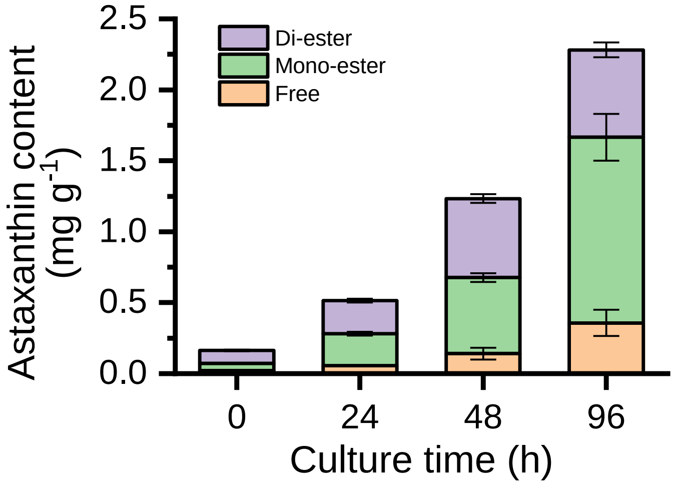

Figure S3. Time course distribution of free astaxanthin, mono-ester, and di-ester in response to 0.2 M NaCl.

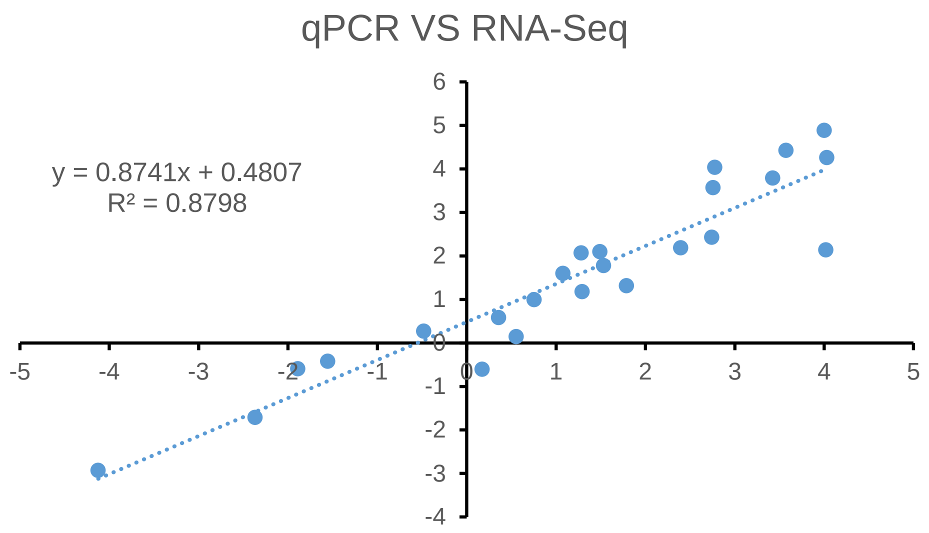

Figure S4. Consistency between RNA-seq–based and qPCR–based transcript quantification. A total of 24 genes were chosen for qPCR validation. The genes and primer sequences for qPCR are listed in Additional file 3: Table S2.

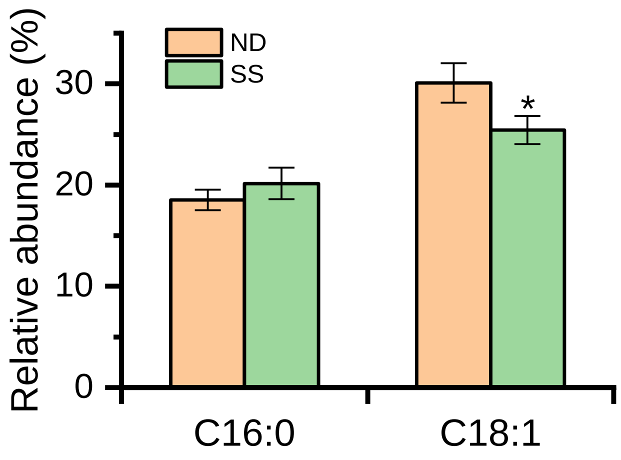

Figure S5. The relative abundance of C16:0 and C18:1 in *sn*-2 position of TAG from *C. zofingiensis* under ND and SS conditions. ND, nitrogen deprivation; SS, 0.2 M salt. Asterisk indicates the significant difference (*t*-test, $p<0.05$) between ND and SS.

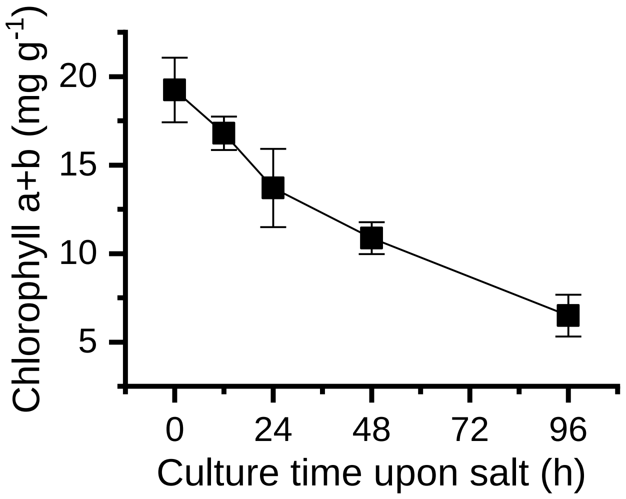

Figure S6. Time course of chlorophyll content in *C. zofingiensis* in the presence of 0.2 M salt.
