# Supplementary material for: Novel insights into salinity-induced lipogenesis and carotenogenesis in the oleaginous astaxanthin-producing alga Chromochloris zofingiensis: a multi-omics study
Source: Biotechnol Biofuels. 2020 Apr 16;13:73. doi: 10.1186/s13068-020-01714-y (PMC7161124; doi:10.1186/s13068-020-01714-y)
Supplement: Supplementary file 3 — Additional file 3: Table S1. Reads quality and mapping ratio of the transcriptomes. Table S2. The primer sequences of selected genes used in qPCR experiments. [file 13068_2020_1714_MOESM3_ESM.pdf]

Table S1 Reads quality and mapping ratio of the transcriptomes

Sample	Clean reads number	Clean reads quality		Mapping ratio (%)		Mapped genes
		Q20 (%)	Q30 (%)	Total	Unique	
SS0-1	23960080	98.35	91.81	97.98	92.76	14682
SS0-2	23636340	97.72	90.13	97.07	91.07	14697
SS0-3	23677786	97.83	90.63	96.88	90.97	14684
SS6-1	23666259	97.97	90.98	97.12	91.22	14233
SS6-2	23778798	98.01	91.04	96.82	91.50	14327
SS6-3	24067181	98.03	90.46	98.26	92.98	14433

SS0 and SS6: 0 h and 6 h upon salinity stress (200 mM NaCl)

Clean Reads Q20(%): The ratio of bases with high quality (Quality value> 20) to the total bases in the clean reads

Clean Reads Q30(%): The ratio of bases with high quality (Quality value > 30) to the total bases in the clean reads

Total Mapping Ratio: The percentage of mapped reads

Uniquely Mapping Ratio: The percentage of reads that map to only one location of reference

Table S2 The primer sequences of selected genes used in qPCR experiments

Gene name	Gene ID	Forward primer (5'-3')	Reverse primer (5'-3')
BC	Cz13g10110	CGTGGCAGCTACGGCTTAAC	TGCACCAACTCCTGGAATGA
KAS3	Cz18g03070	CTGCGGTGCCATAGTGATGA	CCCCCTTCACCGCTGTATTT
KAR	Cz01g34370	TTCATTGCGTCCGACATGAC	AGCTAGGAAGCGCACCAAAC
FAT	Cz04g05080	GAGGGACGGCATGAAATAGC	CAAGTGTGCGGGCATCAC
SAD	Cz04g09090	GATGAGGGACGGCATGAAAT	GTGTGCGGGCATCACAATC
LACS	Cz11g20120	TGGATGACATTGGTGCTCTGA	GCGGGCAAAAGCATAGTTGA
GPAT1	Cz11g03260	GGAGTCTTTGCCCACATGCT	CAGGTTGCGACCCATGCT
GPAT2	Cz09g31330	GCCTTTGCTGTCATCATGCA	GGCAACAATGTGACGATCCTT
LPAAT1	Cz16g02090	GTTCCTCACTGGCCATGTCA	CGTGTGCCTTCTGGGAAGAA
LPAAT2	Cz04g14150	CGTGTGGGATGCAAAATGG	AGCCAGCAAATACCGTCCAA
LPAAT3	Cz10g20070	GGGAACAGCTGCGACTCCTA	CAGTGATGGGCAGGATTGTG
PAP1	Cz05g23060	CACACGCCTGGTCATTTCTG	GCAGGTAGTCGCGAGTGATG
PAP2	Cz10g16040	TGACATCATCGGTGGGTTTG	GTGACAGGCAGGCCAGATCT
PAP3	Cz16g11240	TGGCAGAAGAACCCGAGAAT	CGTGGTGATGCTGTGACGTT
DGAT1A	Cz06g05010	GGCATCCGAATGAACCTCAT	AACTCCTCGTCCTGCTTCATG
DGAT1B	Cz09g08290	TGCCTTGCTTCATCGTCCTT	CCCTTCCCTCCTGGCTACAC
DGTT5	Cz09g27290	CCAGCAGCGTGTTCTCCATT	GCAATACCCCCCACAATCAC
MLDP	Cz04g29220	GCTCCTTCGTTCCTGCATTT	CGCAGCACCTTCTCGCTAGT
LCYe	Cz09g18310	CAGTGTTTCGCGGTCTTTGA	ACGCGTTGGTAGCTGACAGA
LCYb	Cz12g10170	TTCCACTGGCATGGCTTCTT	CTGCCGAGCTCACCATCTG
CHYb	Cz12g16080	CGTTACGCACACAAGGCATT	CACAGGCTGAAGGCAGGTACA
BKT1	Cz13g13100	ACCTCAAGCCGCACTCAAAT	GCCAGCAGCCATGGTAAAAG
bHLH	UNPLg00160	GCTGAGCAACGCAGAAGGA	GAGTTGCGGTGCCTCAATG
MYB	Cz10g24240	CCGCCTGTACCCATGGTAGT	AGCAGTGCAGCAACCAGTTG
Beta-actin	Cz05g19150	GCTGGCATTCACGACACAAC	TGCCACCACCTTGATCTTCA
